# Baseline characteristics and patient reported outcome data of patients prescribed etanercept: web-based and telephone evaluation

**DOI:** 10.1186/1471-2288-11-91

**Published:** 2011-06-14

**Authors:** Alan G Wade, Gordon M Crawford, Neil Pumford, Volker Koscielny, Susan Maycock, Alex McConnachie

**Affiliations:** 1 Patients Direct, Glasgow, UK; 2 Pfizer Ltd, Tadworth, UK; 3 Robertson Centre for Biostatistics, University of Glasgow, Glasgow, UK

## Abstract

**Background:**

The anti-TNF inhibitor, etanercept is administered as a once or twice weekly subcutaneous injection for the treatment of rheumatoid arthritis, psoriasis, ankylosing spondylitis, psoriatic arthritis and juvenile idiopathic arthritis (JIA). Limited data from the patients' perspective are available on the use of biologics in the treatment of these chronic conditions and this evaluation was designed to collect data from patients who had been prescribed etanercept for the first time. This manuscript describes the self-reported baseline characteristics and health-related quality of life of patients prior to treatment. Follow-up data will be reported separately.

**Methods:**

Patients throughout the United Kingdom prescribed etanercept were invited to participate in an evaluation of their condition and treatment using a data collection tool consisting of a web-based system supplemented by telephone reporting (*PROBE*). Outcome measures reported at baseline included demographic data, the condition being treated, previous treatment with biologic agents and current and previous medications. Questions modified from standard, validated quality of life questionnaires such as EQ-5D were incorporated and patients made a global assessment of the severity of their own illness using the CGI-S scale.

**Results:**

A total of 344 patients/carers/parents participated in the evaluation at baseline, 290 (84%) by online questionnaire and 54 (16%) by telephone. Overall, the study population had a mean age of 53 years, was predominantly female (62%) and 20% had been previously treated with a biologic agent. A total of 191 (56%) patients were receiving treatment with etanercept for rheumatoid arthritis, 44 (13%) for psoriatic arthritis, 43 (13%) for ankylosing spondylitis, 35 (10%) for psoriasis, 9 (3%) for known juvenile idiopathic arthritis (JIA) and 22 (6%) for another condition/patient unsure/missing response. All patients were prescribed the 50 mg weekly dose of etanercept except for 1 patient with JIA (40 mg) dose and 2 patients with psoriasis (100 mg). Thirty-eight percent of patients with rheumatoid arthritis were not receiving treatment with methotrexate.

**Conclusions:**

The baseline characteristics and health-related quality of life of first time users of etanercept can be adequately described using self-reported patient data collected using an online questionnaire with a telephone option (*PROBE*).

## Background

Etanercept (Enbrel^®^, Pfizer) is a human tumour necrosis factor alpha (TNF-α) receptor fusion protein produced by recombinant DNA technology. It binds to TNF-α and prevents its interaction with cell surface receptors, thus interfering with the inflammatory cascade. In adults, etanercept can be used to treat active, moderate to severe rheumatoid arthritis, either in combination with methotrexate when the response to standard disease modifying antirheumatic drugs (DMARDs) is inadequate, or as monotherapy, if methotrexate cannot be tolerated or if the disease is severe, active and progressive. Anti-TNF therapies, such as etanercept can slow the rate of progression of joint damage, reduce symptoms such as joint pain, swelling and mobility and improve physical functioning. Etanercept is also used to treat other inflammatory conditions such as psoriasis, ankylosing spondylitis, psoriatic arthritis and juvenile idiopathic arthritis (JIA) in patients who have had an inadequate response to standard therapy. In adults, etanercept is administered as a subcutaneous injection, 50 mg once weekly or 25 mg twice weekly [1].

Observational data on patients treated with anti-TNF therapies for moderate to severe rheumatoid arthritis, ankylosing spondylitis, psoriatic arthritis and other rheumatologic indications are available from the British Society of Rheumatology Biologics Register (BSRBR, [2]). The register captures information recorded by Consultants on a patient's current and previous treatments, disease severity as assessed by American College of Rheumatology (ACR) criteria and current disease activity based on the disease activity score-28 (DAS-28) at baseline. A short form Consultant questionnaire is repeated annually with information collected on events of special interest. Patient-reported outcome data from the EuroQoL [3] and Health Assessment Questionnaire (HAQ, [4]) are collected at baseline and 6-monthly intervals.

Naturalistic research has become increasingly important in assessing the use of therapeutic interventions in clinical practice. Many factors other than those related to the properties of the intervention may influence how it works in a real-life setting, such as the beliefs and behaviour of patients, healthcare providers and general healthcare characteristics.

This evaluation was designed to collect naturalistic data directly from patients who have been prescribed etanercept for the first time using the *PROBE *methodology. *PROBE *has been developed as a self-reported data collection tool by Patients Direct and has been used to collect data in other therapy areas such as side effects following H1N1 and/or seasonal influenza vaccination and for a new product licensed for insomnia. It consists of a web-based system supplemented by telephone reporting for patients preferring this reporting mode. The methodology is relatively inexpensive to administer, allows data to be collected easily from specific target groups and can be tailored to suit different types of investigation. Furthermore, it allows the 'baseline' respondents to be contacted again to provide longitudinal follow-up information, thus enabling patient-reported outcomes on the effectiveness, healthcare experience, adverse events and persistence of etanercept treatment to be tracked.

Electronic collection of patient-reported outcome data using web-based technology is becoming an established way of gathering health data. However, patients who respond on the Internet may not be typical of the general population, potentially being younger, more educated and of a higher social class. By providing the alternative option of participating using a Freephone service, the *PROBE *methodology alleviates any problem that the elderly or other groups may have in accessing the Internet and allows a more representative population to be obtained, which is vital for reducing bias. Original data used to perform this analysis is provided in Additional file 1.

In this evaluation, the research methodology of *PROBE *differs from that of the BSRBR as it is entirely patient (or carer/parent) reported and has a wider scope. Post-baseline data were collected on a patient's healthcare experience measured through questions such as time between prescription and delivery of etanercept and quality of injection training, and there were additional quality of life measures.

The overall aims of the evaluation were:

• To determine the baseline characteristics of patients prescribed etanercept

• To evaluate the healthcare experience of patients prescribed etanercept by assessing the service provision from hospital specialists through to home care delivery and training.

• To measure patient-reported outcomes including persistence with treatment, benefits of treatment and adverse events

In this manuscript, a full description of the *PROBE *methodology is given under "methods" below and the baseline data are reported. Data on the healthcare experience of patients prescribed etanercept and follow-up data will be reported in separate manuscripts.

## Methods

### Study design

This evaluation was designed to collect naturalistic data directly from patients prescribed etanercept using the *PROBE *methodology consisting of a web-based system supplemented by telephone reporting. It was conducted throughout the United Kingdom by Patients Direct, Glasgow.

### Patients

Patients who had been newly prescribed etanercept by their specialist between April and November 2009 were invited to participate in the evaluation through leaflets. These were distributed to approximately 1000 patients. The initial supply of etanercept was delivered by a company providing nursing services in the patient's home. At the time of delivery, patients were provided with an invitation letter and a written information sheet containing detailed information about the evaluation including the website address of Patients Direct (http://www.patientsdirect.net) and a 4-digit PIN code specific to each patient. In addition, patients were given a support pack designed by Wyeth/Pfizer, which contained written materials and an instructional DVD to educate patients about the correct way to use etanercept. The project and methodology were submitted to the National Research Ethics Centre (NRES). They confirmed that this project did not require formal approval.

### Patient Reported Outcomes Based Evaluation (*PROBE*)

#### Web-based mode

Patients using the web-based mode of reporting logged onto the secure website via the Enbrel section of http://www.patientsdirect.net by entering the PIN code. They were then prompted to choose a personal password. Consent was obtained electronically and consenting patients were asked to answer a questionnaire, which took approximately 10 minutes to complete. Questions were displayed in a logical sequence using radio buttons, check boxes, drop down menus and free text for responses. Many questions also had the option of 'I am not sure'. The layout of the online questionnaire included 'smart branding' to switch to the next relevant question and reduce complexity. For example, only patients with known rheumatoid arthritis were asked about their DAS-28. A summary of the information collected at baseline is presented in Table 1. Patients were asked to complete the baseline questions before they had started treatment with Enbrel. If they had already started treatment, they were instructed to answer the questions as if it was before they had started treatment.

**Table 1:** Outcome measures collected at baseline

• Demographical details (first name, age, sex, E-mail, first part of post code)
• Condition being treated
• Previous treatment with biologic agents
• Current medications including methotrexate
• Functional status and general quality of life
- CGI-S
- EQ-5D and EQ VAS
• Disease-specific quality of life
- HAQ for patients with rheumatoid arthritis
- CHAQ for patients with juvenile idiopathic arthritis (JIA)
- DSLI for patients with psoriasis
• DAS-28 for patients with rheumatoid arthritis

CGI-S Clinical Global Impression - Severity of Illness, CHAQ Child Health Assessment Questionnaire, DAS-28 Disease Activity Score-28, DSLI Dermatology Life Quality Index, EQ-5D EuroQol 5 dimensions, EQ VAS, EuroQol visual analogue scale, HAQ Health Assessment Questionnaire

#### Telephone reporting

Patients without Internet access or those who preferred to use the telephone were able to participate in the evaluation by using a Freephone number and speaking to a research nurse. Verbal consent was obtained. The research nurse went through the questions in the same structured format, with visual analogue scales (VAS) described by stating what the minimum and maximum values represented and asking patients where they felt they were on the scale. All responses were entered directly into the web-based database. The research nurse also provided telephone support to Internet users.

#### Follow-up data

The flexibility of the *PROBE *system allowed additional data to be collected. After 2 weeks, 1 month and monthly intervals up to 6 months, patients were contacted by E-mail, text message or telephone and reminded to revisit the website to answer further questions or to respond by telephone. An information and consent page preceded collection of effectiveness (patient symptoms), side effect and persistence data. No longitudinal follow-up data are presented in this manuscript.

A lower proportion of patients with rheumatoid arthritis reported concomitant use of methotrexate at baseline than expected. Therefore, patients responding "no" to the question "Are you taking methotrexate" were contacted to obtain further information on any previous use of methotrexate and if relevant, their reason for stopping treatment.

This requirement to contact patients to verify their answers could not easily be foreseen in setting up the project and illustrates the flexibility of the methodology. Functional status and Quality of Life Instruments.

### Functional status and Quality of Life Instruments

Electronic facsimiles of the following functional status and quality of life instruments were used in the evaluation. All patients were asked Clinical Global Impressions-Severity of Illness (CGI-S) scale and EuroQol 5 dimensions (EQ-5D) questions. Patients with rheumatoid arthritis, JIA or psoriasis were also asked more disease specific questions from the HAQ, Child Health Assessment Questionnaire (CHAQ) and Dermatology Life Quality Index (DLQI) instruments respectively.

#### Clinical Global Impressions-Severity of Illness (CGI-S)

Functional status of all patients was assessed using the CGI-S scale, which is a 7-point scale ranging from 1 = normal to 7 = extremely ill [5]. This self-assessed evaluation of patients' overall condition was in response to the question: "Overall how bad do you consider your condition now?

#### EuroQol 5 dimensions (EQ-5D)

Quality of life was assessed in all patients using the EQ-5D descriptive system and the EQ visual analogue scale (EQ VAS) questions developed by the EuroQol group [3]. The EQ-5D descriptive system assesses 5 dimensions (mobility, self-care, usual activities, pain/discomfort and anxiety/depression) on a 3-point scale (1 = no problems, 2 = some problems, 3 = severe problems). Patients indicated which statements best described their health state on that day for each of the 5 dimensions. A weighted health status index score was calculated for the data by applying scores from the appropriate available 'value sets'. A higher score indicates a better quality of life, with a maximum value of 1, and zero represents a health state equivalent to death. The EQ VAS records a patient's health state on a visual analogue scale where the endpoints range from 'best imaginable health state' (100) to 'worst imaginable health state' (0). The conventional, paper-based version of the EQ VAS uses a vertical alignment similar to a thermometer. However, in the web-based assessment, a horizontal visual analogue scale was used and patients used a pointer to indicate how good or bad their health state was on that day. In the telephone assessment, a verbal description of the scale was given and patients asked to provide a number representing their health state. Both the web-based and telephone reporting modes used the same wording for the endpoints.

#### Health Assessment Questionnaire (HAQ)

The HAQ disability index (HAQ-DI) was used to assess the level of disability in patients with rheumatoid arthritis. It is based on 20 items in 8 domains: dressing, rising, eating, walking, hygiene, reach, grip and usual activities and includes questions on the use of aids or devices and whether assistance is needed from another person [4]. Each item has 4 levels ranging from 0 = without any difficulty to 3 = unable to do, with standard scoring taking the use of aids and devices or assistance from another person into account. The mean of the domain scores gives the disability index, which varies from 0 to 3 points (highest degree of incapacity).

#### Child Health Assessment Questionnaire (CHAQ)

The CHAQ was used to assess the functional ability of children and adolescents with JIA to perform daily living activities [6]. It is made up of 30 items in the same 8 domains as the HAQ-DI and uses a similar scoring system. 'Not applicable' is available as an additional option for each item as some items could not be applied to young children. The CHAQ VAS Pain Scale question was included to assess the severity of pain related to the illness over the past week and was scored from 0 (no pain) to 100 (very bad pain).

#### Dermatology Life Quality Index (DLQI)

The DLQI is a dermatology-specific instrument consisting of 10 questions concerning the effect of the skin problem on a patient's quality of life over the previous week [7].

### Sample size and statistical methods

No formal sample size calculations were conducted prior to the study, since the objective of the research was exploratory, and data were being collected in a novel manner with no prior knowledge of likely uptake rates. However, to illustrate the value of the data, a sample size of 300 respondents provides sufficient information to estimate prevalence figures with a 95% confidence interval of no more than ±5.7%; within subgroups of 100 individuals, this precision is ±9.8%.

Statistical analyses were performed using Splus for Windows v8.1. Baseline patient characteristics and responses have been summarised descriptively. Post hoc analyses were performed to compare the baseline characteristics of Internet and telephone users using the Wilcoxon-Mann-Whitney test and to compare the CGI-S and EQ-5D measures by the condition treated using both the Kruskal-Wallis test and Analysis of Variance (ANOVA).

## Results

### Patient disposition

**Figure 1:**
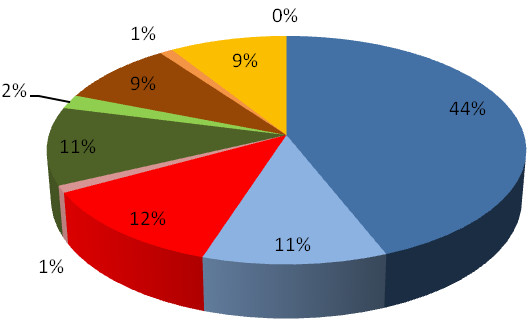
**Pie chart of patients participating in the evaluation, by condition and reporting mode**. Percentages are given to the nearest whole number. Blue: Rheumatoid arthritis - Internet Light blue: Rheumatoid arthritis - Telephone Red: Ankylosing spondylitis - Internet Pink: Ankylosing spondylitis - Telephone Dark green: Psoriatic arthritis - Internet Light green: Psoriatic arthritis - Telephone Brown: Psoriasis - Internet Orange: Psoriasis - Telephone Yellow: Juvenile idiopathic arthritis/other - Internet No colour: Juvenile idiopathic arthritis/other - Telephone.

A total of 344 patients treated with etanercept responded to the invitation to participate in the baseline evaluation between April 2009 and April 2010, with 290 (84%) using the web-based mode and 54 (16%) using the telephone (Table 2). The breakdown by reporting mode and condition is shown in Figure 1. The rough estimate of uptake was 34% based on distribution of about 1,000 leaflets and assuming one leaflet per patient. However, the actual number of patients who were eligible to participate is unknown. The respondents consisted of patients (n = 331), carers (n = 6) and parents (n = 7). There was a high response rate to questions at baseline. The average response rate for questions asked of all respondents was 95% (range 93% to 100%), excluding the EQ-5D VAS question which was completed by only 46%. Original data used to perform this analysis is provided in Additional file 2.

**Table 2:** Baseline characteristics of participants, by reporting mode

	Internet	Telephone	Total
Completed, n (% of total)	290 (84.3%)	54 (15.7%)	344 (100%)
Completed by: n (%)			
Patient	277 (95.5%)	54 (100%)	331 (96.2%)
Carer	6 (2.1%)	0 (0%)	6 (1.7%)
Parent	7 (2.4%)	0 (0%)	7 (2.0%)
Age, mean years (range)	51.4 (13-77)^a^	61.5 (35-82)	53.1 (13-82)
Sex, n (%) M/F	103/166 (38.3/61.7)	19/35 (35.2/64.8)	122/201 (37.8/62.2)
Diagnosis, n (%) [mean age, %F]			
Rheumatoid arthritis	152 (79.6%) [53.9, 72%]	39 (20.4)% [64.4, 67%]	191 (100%)[56.0, 71%]
Psoriasis	31 (88.6%) [48.2, 52%]	4 (11.4%) [61.2, 75%]	35 (100%) [49.8, 62%]
Ankylosing spondylitis	40 (93.0%)[49.1, 25%]	3 (7.0%) [51.3, 33%]	43 (100%) [49.2, 26%]
Psoriatic arthritis	37 (84.1%) 50.3, 68%]	7 (15.9%) [51.9, 71%]	44 (100%) [50.5, 68%]
Other^b^	30 (96.8%) [41.7, 63%]	1 (3.2%) [48.0, 0%]	31 (100%) [42.1, 62%]
Previous biologics			
N	275	54	329
Any	59 (21.5%)	6 (11.1%)	65 (19.8%)
Adulimumab	43 (15.6%)	5 (9.3%)	48 (14.6%)
Infliximab	11 (4.0%)	1 (1.9%)	12 (3.6%)
Current medications			
N	277	54	331
Methotrexate	120 (43.3%)	29 (53.7%)	149 (45.0%)
Sulfasalazine	46 (16.6%)	7 (13.0%)	53 (16.0%)
Prednisolone	55 (19.9%)	14 (25.9%)	69 (20.8%)
Quality of life			
CGI-S			
N	275	54	329
N ( %) ≥markedly ill)	184 (66.9%)	30 (55.6%)	214 (65.0%)
EQ-5D			
N	265	54	(0.35)
EQ5D Index, Mean (SD)	0.41 (0.36)	0.45 (0.29)	0.42
N	139	20	159
EQ5D VAS, Mean (SD)	47.6 (23.0)	51.2 (21.8)	48.1 (22.8)
HAQ			
N	151		190
Mean (SD)	1.68 (0.64)^c^	1.97	1.74 (0.65)
DLQI			
N	31	4	35
Mean (SD)	15.5 (8.1)	11.8 (3.4)	15.0 (7.8)

^a ^Statistically significant difference between Internet and telephone users, p < 0.001 (Wilcoxon test)

^b ^Includes patients with juvenile idiopathic arthritis (n = 9, 2.6%), patients responding other or not sure of condition (n = 10, 2.9%) and patients with condition missing (n = 12, 3.5%) to the question 'What condition do you have that is being treated with Enbrel?'

^c ^Statistically significant difference between Internet and telephone users, p = 0.003 (Wilcoxon test), p = 0.010 (t-test)

### Patient characteristics and conditions

The baseline characteristics of the patients are given in Tables 2 and 3, by reporting mode and condition respectively. The majority of patients (191, 56%) were receiving treatment with etanercept for rheumatoid arthritis, with 35 (10%) patients receiving treatment for psoriasis, 44 (13%) for psoriatic arthritis, 43 (12%) for ankylosing spondylitis and 9 (3%) for known JIA. In addition, 10 (2.9%) patients had another condition or were unsure of their condition and 12 (3.5%) patients had missing data for this question. The patients had a mean age of 53 years and were predominantly female (201/323, 62%), which was expected due to the higher incidence of rheumatoid arthritis in females.

**Table 3:** Demographic characteristics of participants completing the baseline evaluation, by condition

	Rheumatoid arthritis	Ankylosing spondylitis	Psoriatic arthritis	Psoriasis	**Other **^**a**^	Total
Participants, n (% of total)	191 (55.5%)	43 (12.5%)	44 (12.8%)	35 (10.2%)	31 (9.0%)	344 (100%)
Patient, n (%)	189 (99.0%)	43 (100.0%)	41 (93.2%)	33 (94.3%)	25 (80.6%)	331 (96.2%)
Carer, n (%)	2 (1.0%)	0 (0.0%)	2 (4.5%)	1 (2.9%)	1 (3.2%)	6 (1.7%)
Parent, n (%)	0 (0.0%)	0 (0.0%)	1 (2.3%)	1 (2.9%)	5 (16.1%)	7 (2.0%)
Sex						
N [N missing]	189 [2]	43 [0]	41 [3]	33 [2]	17 [14]	323 [21]
Male, n (%)	55 (29.1%)	32 (74.4%)	13 (31.7%)	15 (45.5%)	7 (41.2%)	122 (37.8%)
Female, n (%)	134 (70.9%)	11 (25.6%)	28 (68.3%)	18 (54.5%)	10 (58.8%)	201 (62.2%)
Age						
N [N missing]	189 [2]	43 [0]	41 [3]	33 [2]	17 [14]	323 [21]
Mean (SD)	56.0 (12.5)	49.2 (12.3)	50.5 (9.5)	49.8 (11.0)	42.1 (13.4)	53.1 (12.6)
Range	20.0-79.0	27.0-71.0	34.0-68.0	27.0-82.0	13.0- 68.0	13.0-82.0

^a ^Includes patients with juvenile idiopathic arthritis (n = 9, 2.6%), patients responding other or not sure of condition (n = 10, 2.9%) and patients with condition missing (n = 12, 3.5%) to the question 'What condition do you have that is being treated with Enbrel?'

There were some differences in the baseline characteristics of respondents using the Internet and telephone. The mean age of patients completing the evaluation by telephone was significantly higher than Internet respondents (61.5 y vs. 51.4 y, p < 0.001). Patients with rheumatoid arthritis responding using the telephone were significantly older (64.4 y vs. 53.9 y, p < 0.001) and also had a significantly higher level of disability than those using the Internet, as assessed by the HAQ (1.68 vs. 1.97, p = 0.003).

### Medications

#### Previous use of biologic agents

One fifth of patients participating in the evaluation had been previously treated with a biologic agent. The TNF inhibitor, adalimumab was the most commonly used biologic (Table 4). Some patients (12%) were unsure whether or not they had been treated with a biologic. No patients had previously been treated with etanercept as the baseline evaluation was carried out in patients following their first prescription.

**Table 4:** Number (%) of patients with prior treatment of a biologic agent

	Rheumatoid arthritis	Ankylosing spondylitis	Psoriatic arthritis	Psoriasis	**Other **^**a**^	Total
N [N missing]	188 [3]	43 [0]	44 [0]	35 [0]	19 [12]	329 [15]
No prior treatment	127 (67.6%)	28 (65.1%)	30 (68.2%)	24 (68.6%)	15 (78.9%)	224 (68.1%)
Unsure of prior treatment	24 (12.8%)	5 (11.6%)	6 (13.6%)	4 (11.4%)	1 (5.3%)	40 (12.2%)
Any prior treatment	37 (19.7%)	10 (23.3%)	8 (18.2%)	7 (20.0%)	3 (15.8%)	65 (19.8%)
Adalimumab	31 (16.5%)	5 (11.6%)	7 (15.9%)	2 (5.7%)	3 (15.8%)	48 (14.6%)
Infliximab	7 (3.7%)	4 (9.3%)	0 (0.0%)	1 (2.9%)	0 (0.0%)	12 (3.6%)
Anakinra	2 (1.1%)	0 (0.0%)	0 (0.0%)	0 (0.0%)	0 (0.0%)	2 (0.6%)
Abatacept	0 (0.0%)	1 (2.3%)	0 (0.0%)	0 (0.0%)	0 (0.0%)	1 (0.3%)
Rituximab	3 (1.6%)	1 (2.3%)	1 (2.3%)	0 (0.0%)	0 (0.0%)	5 (1.5%)
Efalizumab	1 (0.5%)	0 (0.0%)	0 (0.0%)	4 (11.4%)	0 (0.0%)	5 (1.5%)

^a ^Includes patients with juvenile idiopathic arthritis (n = 9, 2.6%), patients responding other or not sure of condition (n = 10, 2.9%) and patients with condition missing (n = 12, 3.5%) to the question 'What condition do you have that is being treated with Enbrel?'

#### Methotrexate use

The current use of methotrexate and other medications is given in Tables 5 and 6 respectively. Methotrexate was the most commonly taken second-line therapy for all conditions (62% rheumatoid arthritis, 36% psoriatic arthritis, 11% psoriasis, 42% other) except for ankylosing spondylitis (7%), for which prednisolone was more commonly used (14%). The reasons for patients not taking methotrexate were investigated further by contacting patients who were not taking it at baseline. Overall, 57% of these patients provided feedback. The most commonly reported reason for stopping methotrexate treatment was given as 'side effects' (71%), followed by 'lack of effect' (16%) and 'stopped by doctor' (12%).

**Table 5:** Use of methotrexate at baseline

	Rheumatoid arthritis	Ankylosing spondylitis	Psoriatic arthritis	**Psoriasis **^**a**^	**Other **^**b**^	Total
N [N missing]	190 [1]	43 [0]	44 [0]	35 [0]	19 [12]	331 [13]
Taking methotrexate						
No, n (%)	71 (37.4%)	38 (88.4%)	27 (61.4%)	31 (88.6%)	11 (57.9%)	178 (53.8%)
Unsure, n (%)	1 (0.5%)	2 (4.7%)	1 (2.3%)	0 (0.0%)	0 (0.0%)	4 (1.2%)
Yes, n (%)	118 (62.1%)	3 (7.0%)	16 (36.4%)	4 (11.4%)	8 (42.1%)	149 (45.0%)
Route of administration						
Tablets, n (%)	97 (82.2%)	3 (100.0%)	11 (68.8%)	3 (75.0%)	6 (75.0%)	120 (80.5%)
Injection, n (%)	21 (17.8%)	0 (0.0%)	5 (31.2%)	1 (25.0%)	2 (25.0%)	29 (19.5%)
Weekly dose						
N [N missing]	116 [2]	3 [0]	16 [0]	4 [0]	7 [1]	146 [3]
Mean (SD)	16.6 (6.8)	17.5 (2.5)	15.6 (7.3)	16.2 (9.7)	13.6 (8.9)	16.4 (6.9)
Range	2.5-30.0	15.0-20.0	2.5-25.0	2.5-25.0	2.5-25.0	2.5-30.0
Feedback received						
Yes, n (% of not taking)	40 (56.3%)	21 (55.3%)	19 (70.4%)	16 (51.6%)	5 (45.5%)	101 (56.7%)
Ever prescribed ^c^						
No, n (%)	1 (2.5%)	18 (85.7%)	0 (0.0%)	5 (31.2%)	1 (20.0%)	25 (24.8%)
Unsure, n (%)	1 (2.5%)	1 (4.8%)	0 (0.0%)	1 (6.2%)	0 (0.0%)	3 (3.0%)
Yes, n (%)	38 (95.0%)	2 (9.5%)	19 (100.0%)	10 (62.5%)	4 (80.0%)	73 (72.3%)
Reason stopped ^d^						
Lack of effect, n (%)	6 (15.8%)	1 (50.0%)	1 (5.3%)	4 (40.0%)	0 (0.0%)	12 (16.4%)
Side effects, n (%)	27 (71.1%)	1 (50.0%)	16 (84.2%)	5 (50.0%)	3 (75.0%)	52 (71.2%)
Doctor stopped, n (%)	5 (13.2%)	0 (0.0%)	2 (10.5%)	1 (10.0%)	1 (25.0%)	9 (12.3%)
Unsure, n (%)	0 (0.0%)	0 (0.0%)	0 (0.0%)	0 (0.0%)	0 (0.0%)	0 (0.0%)

^a ^Twenty-five patients (71.4%) with psoriasis were also using creams for the condition at baseline, but none of the patients had undergone phototherapy treatment.

^b ^Includes patients with juvenile idiopathic arthritis (n = 9, 2.6%), patients responding other or not sure of condition (n = 10, 2.9%) and patients with condition missing (n = 12, 3.5%) to the question 'What condition do you have that is being treated with Enbrel?

^c ^Data for feedback responders (Respondents answering "no" to the question " Are you taking methotrexate" at baseline and providing feedback on any previous use)

^d ^Data for feedback responders ever prescribed methotrexate (Feedback respondents answering "yes" to the question " Have you ever been prescribed methotrexate")

**Table 6:** Current medications other than methotrexate used to treat condition at baseline

	Rheumatoid arthritis	Ankylosing spondylitis	Psoriatic arthritis	Psoriasis	**Other **^**a**^	Total
N [N missing]	190 [1]	43 [0]	44 [0]	35 [0]	19 [12]	331 [13]
Sulfasalazine, n (%)	41 (21.6%)	4 (9.3%)	6 (13.6%)	0 (0.0%)	2 (10.5%)	53 (16.0%)
Leflunomide, n (%)	14 (7.4%)	0 (0.0%)	4 (9.1%)	0 (0.0%)	0 (0.0%)	18 (5.4%)
Prednisolone, n (%)	53 (27.9%)	6 (14.0%)	5 (11.4%)	1 (2.9%)	4 (21.1%)	69 (20.8%)
Ciclosporin, n (%)	2 (1.1%)	0 (0.0%)	1 (2. 3%)	1 (2.9%)	1 (5.3%)	5 (1.5%)
Acitretin, n (%)	3 (1.6%)	0 (0.0%)	0 (0.0%)	2 (5.7%)	0 (0.0%)	5 (1.5%)

N [N missing]	-	-	-	35 [0]	-	35 [309]
Creams for psoriasis	-	-	-	25 (71.4%)	-	25 (71.4%)
Phototherapy	-	-	-	0 (0%)	-	0 (0%)

^a ^Includes patients with juvenile idiopathic arthritis (n = 9, 2.6%), patients responding other or not sure of condition (n = 10, 2.9%) and patients with condition missing (n = 12, 3.5%) to the question 'What condition do you have that is being treated with Enbrel?

#### Other medications

Prednisolone and sulfasalazine were commonly taken to treat all the conditions other than psoriasis (Table 6). Patients with psoriasis mainly used creams for the condition at baseline (71%) and none of the patients had undergone phototherapy treatment.

#### Etanercept dose

The mean weekly dose of etanercept prescribed was 50 mg (range 40 to 100 mg); the 40 mg dose was used in 1 patient with JIA and 100 mg was used by 2 patients with psoriasis.

### Functional status and quality of life

The CGI-S responses indicated that 65% of patients (214/329) were at least markedly ill at baseline (Table 7). The general quality of life (EQ-5D Index and VAS scores) and more disease specific measures (CHAQ, DLQI, HAQ) are also shown in Table 6.

**Table 7:** Number (%) of patients with functional status and quality of life measures at baseline

Score	Rheumatoid arthritis	Ankylosing spondylitis	Psoriatic arthritis	Psoriasis	**Other **^**a**^	Total
**CGI-Severity**						
N [N missing]	190 [1]	43 [0]	42 [2]	35 [0]	19 [12]	329 [15]
Extremely ill	19 (10.0%)	0 (0.0%)	3 (7.1%)	5 (14.3%)	4 (21.1%)	31 (9.4%)
Severely ill	39 (20.5%)	15 (34.9%)	3 (7.1%)	8 (22.9%)	4 (21.1%)	69 (20.9%)
Markedly ill	66 (34.7%)	18 (41.9%)	17 (40.5%)	10 (28.6%)	3 (15.8%)	114 (34.7%)
Moderately ill	45 (23.7%)	7 (16.3%)	14 (33.3%)	5 (14.3%)	4 (21.1%)	75 (22.8%)
Mildly ill	13 (6.8%)	3 (7.0%)	3 (7.1%)	4 (11.4%)	2 (10.5%)	25 (7.6%)
Borderline	4 (2.1%)	0 (0.0%)	2 (4.8%)	3 (8.6%)	1 (5.3%)	10 (3.0%)
Normal	4 (2.1%)	0 (0.0%)	0 (0.0%)	0 (0.0%)	1 (5.3%)	5 (1.5%)
Mean (SD) ^**b**^	3.12 (1.28)	2.95 (0.90)	3.40 (1.13)	3.11 (1.49)	3.16 (1.77)	
**EQ VAS**						
N [N missing]	85 [106]	22 [21]	25 [19]	21 [14]	6 [25]	159 [185]
Mean (SD) ^**b**^	49.5 (22.4)	42.2 (23.8)	45.6 (19.2)	50.7 (25.6)	50.5 (30.8)	48.1 (22.8)
Range	8.0-100.0	6.0-90.0	8.0-85.0	5.0-90.0	20.0-92.0	5.0-100.0
**EQ-5D Index**						
N [N missing]	181 [10]	43 [0]	43 [1]	34 [1]	18 [13]	319 [25]
Mean (SD) ^c^	0.40 (0.34)	0.37 (0.37)	0.42 (0.32)	0.52 (0.39)	0.51 (0.40)	0.42 (0.35)
Range	-0.24-1.00	-0.24-1.00	-0.24-0.80	-0.48-1.00	-0.35-1.00	-0.48-1.00

**Disease specific**	**HAQ Score**			**DLQI Score**	**CHAQ Score**	
N [N missing]	190 [1]	-	-	35 [0]	9 [22]	
Mean (SD)	1.74 (0.65)	-	-	15.0 (7.8)	1.35 (0.54)	
Range	0.00-3.00	-	-	3.0-30.0	0.50-2.25	
					**CHAQ Pain Score**	
N [N missing]	-	-	-	-	8 [23]	
Mean (SD)	-	-	-	-	49.1 (30.1)	
Range	-	-	-	-	7.0-90.0	

^a ^Includes patients with juvenile idiopathic arthritis (n = 9, 2.6%), patients responding other or not sure of condition (n = 10, 2.9%) and patients with condition missing (n = 12, 3.5%) to the question 'What condition do you have that is being treated with Enbrel?

b No significant differences in mean CGI-S or EQ-5D VAS scores between conditions using the Kruskal-Wallis test or ANOVA.

^c ^No significant differences in mean CGI-S or EQ-5D VAS scores between conditions using the Kruskal-Statistically significant differences in EQ-5D Index scores between the conditions using the Kruskal-Wallis test (p = 0.043), but not with ANOVA, p = 0.259.

CHAQ Child Health Assessment Questionnaire (for patients with juvenile idiopathic arthritis), CGI Clinical Global Impression, DLQI Dermatology Life Quality Index (for patients with psoriasis), EQ EuroQoL, HAQ Health Assessment Questionnaire (for patients with rheumatoid arthritis), VAS Visual analogue scale

Patients with ankylosing spondylitis appear to rate their quality of life as being worse than other patients taking etanercept. This was in agreement with the numerically lower mean CGI-S score of patients with ankylosing spondylitis than those with other conditions. However, whilst there was some evidence of a difference in quality of life between the conditions, as assessed by the mean EQ 5D Index scores (p = 0.043, Kruskal -Wallis test, p = 0.259, ANOVA), there was no significant difference between the CGI-S scores.

### Disease Activity Score-28 (DAS-28)

Over 90% of patients (172/190) with rheumatoid arthritis did not know their DAS-28 score. Of those who stated they did know their score, clearly many did not understand or correctly recall it as many gave values greater than 10 (mean 27.06, SD 36.96, range 4-105).

## Discussion

Patient-reported outcome data are becoming extensively used not only in clinical trials and epidemiological studies, but also in assessing patient care and adverse event reporting [8-12]. This evaluation describes the use of *PROBE *as a method for gathering information on patients prescribed a biological DMARD. It showed that an online questionnaire with a telephone option available for respondents was an effective way of collecting self-reported patient data. The importance of providing telephone support was indicated by 16% of respondents using this reporting mode. Telephone respondents generally had a similar baseline profile to Internet respondents, but were significantly older. This was an expected finding, as although Internet access is increasing in the elderly, this group still lags behind other age groups in Internet usage [13]. Anecdotal evidence also suggested that many respondents required telephone reassurance to lead them through the initial questions, but thereafter reverted to self-completion. Hence, telephone access was important in helping to gain a representative population of first time users of etanercept. Although the respondents were mainly the patients themselves, a small proportion (3%), was the patient's parent or carer, highlighting that information can be gathered successfully from other parties when relevant.

The evaluation was conducted in 344 patients throughout the United Kingdom who had been prescribed etanercept, with the geographical spread of patients confirmed by the first part of their postcode. As expected due to the conditions under treatment, the majority of the patients (69% with sex recorded) who participated in the evaluation were women.

Patients with rheumatoid arthritis had a mean age of 55.9 years and 70.5% were women. This closely matched the characteristics recently reported in an epidemiological study with 466 patients (mean age 55.6 years, 69% women, [14]). It is reassuring that the population captured in this evaluation using Internet technology and telephone reporting reflects that of the existing literature. However, there was a significant difference between the baseline characteristics of those responding by telephone and the Internet, with those responding by telephone significantly older and more disabled by the condition. Again, this illustrates the importance of the telephone reporting system in limiting bias by widening participation and enabling information to be gathered from patients who may lack Internet access.

The CGI-S responses of patients with rheumatoid arthritis showed that 89% considered themselves at least moderately ill. This reflected responsible prescribing with etanercept given according to the license for the treatment of moderate to severe rheumatoid arthritis. However, the proportion of patients with rheumatoid arthritis not receiving treatment with methotrexate was considerably higher than expected (38%). Feedback requested from patients not currently taking methotrexate had shown that side effects were the main reason for stopping treatment. The majority of patients contacted for additional feedback responded illustrating their positive interaction with Patients Direct and the potential for obtaining further information in this manner.

It is also worth noting that very few patients with rheumatoid arthritis and prescribed etanercept were aware of their DAS-28 score, which needs redressing if patients are to take a more active role in managing their own healthcare.

National Institute of Clinical Excellence (NICE) guidelines (TA130), which were in place during the evaluation stated that patients with rheumatoid arthritis would not be able to try a second TNF inhibitor if their first attempt failed, unless therapy was withdrawn due to an adverse event [15]. NICE have since issued guidelines (TA195, 2010) allowing the use of a second TNF inhibitor [16]. As 21% of patients participating in the evaluation had previously received treatment with adalimunab or infliximab, this would appear to indicate that switching to a second TNF inhibitor was accepted practice before the update from NICE.

### Limitations

There are a number of limitations with the evaluation. One of the limitations is that the number of patients eligible to participate is unknown as the precise number of leaflets distributed is only a rough estimate. Hence, the estimated uptake rate of 34% is imprecise and this may result in recruitment bias between patients responding to the invitation and non-responders.

Internet usage is associated with various socioeconomic and demographic factors including age, sex, location and education [13]. Hence, a disadvantage with data collected using web technology is the tendency for users not to be representative of the target population. This is largely overcome by having a telephone option. However, there may be some disadvantages from grouping together these two modes of reporting as there may be differences in responses between telephone and Internet respondents. Questions were completed more fully and the mean age was higher in respondents using the telephone option rather than the Internet. It is also known that certain side effects such as emotional distress and problems with sexual function are less likely to be reported as side effects over the telephone compared with the Internet. Questions concerning unusual symptoms affecting sexual function were asked at the follow up time points, but were not part of the baseline assessment.

There may also be some concerns about grouping reports from patients with those of their parents or carers, although these represented only a small contribution of the overall sample size (4%). Their inclusion allowed information to be collected on young children with JIA and patients more disabled by their condition. The authors felt that including parent/carer reported data in the evaluation would lead to less bias than from their exclusion.

The user satisfaction and acceptability of *PROBE *and ease of responding to the questions asked should also have been assessed.

## Conclusions

The baseline characteristics and health-related quality of life of first time users of etanercept can be adequately described using self-reported patient data collected using an online questionnaire with a telephone option (*PROBE*).

Baseline data obtained from patients with rheumatoid arthritis using this methodology are similar to those reported in the literature. Self-reported data indicates that patients with rheumatoid arthritis need to better informed of their DAS-28 score and that their concomitant use of methotrexate is lower than expected.

## Competing interests

AW has no competing interests. He is a Director of the company "Patients Direct" which has been funded by Pfizer to setup and undertake the work described in this manuscript.

GC has no competing interests. He is a Director of the company "Patients Direct" which has been funded by Pfizer to setup and undertake the work described in this manuscript.

NP is an employee of Patients Direct.

VK is an employee of Pfizer UK.

SM is an employee of Pfizer UK.

AMcC has no competing interests.

## Authors' contributions

AW was involved in the conception, design, set up, acquisition of data, analysis and interpretation of data, reviewing drafts of the manuscript and approval of final version. GC was involved in the conception, design, set up, acquisition of data, analysis and interpretation of data, reviewing drafts of the manuscript and approval of final version. NP was involved in acquisition of data, data verification, designing data reports, analysis and interpretation of data, designing the manuscript content, reviewing drafts of the manuscript and approval of final version. VK was involved in the conception, design, set up, interpretation of data, reviewing drafts of the manuscript and approval of final version. SM was involved in the conception, design, set up, interpretation of data, reviewing drafts of the manuscript and approval of final version. AMcC was involved in the data extraction, data cleaning, designing data reports, analysis and statistical interpretation of data, reviewing drafts of the manuscript and approval of final version.

## Pre-publication history

The pre-publication history for this paper can be accessed here:

http://www.biomedcentral.com/1471-2288/11/91/prepub

## Supplementary Material

Additional File 1 **Word document Breakdown of telephone, internet and all responses on baseline characteristics**. Click here for file

Additional File 2 **Word document Breakdown of question completion by telephone, internet and all respondents**. Click here for file
