# Supplementary material for: Baseline characteristics and patient reported outcome data of patients prescribed etanercept: web-based and telephone evaluation
Source: BMC Med Res Methodol. 2011 Jun 14;11:91. doi: 10.1186/1471-2288-11-91 (PMC3141801; doi:10.1186/1471-2288-11-91)
Supplement: Additional File 1 — Word document Breakdown of telephone, internet and all responses on baseline characteristics. [file 1471-2288-11-91-S1.DOC]

**Additional File 1**

| **Table 3.1.1:** Age, sex and who completed the questionnaire. Sex-specific figures exclude subjects with no Age or Sex information. | | | | | | | | | | | | |
| --- | --- | --- | --- | --- | --- | --- | --- | --- | --- | --- | --- | --- |
|  |  | All |  | Males | | | |  | Females | | | |
|  |  |  |  | All | < 40 | 40-59 | ≥ 60 |  | All | < 40 | 40-59 | ≥ 60 |
| Age (years) | N [N_MISSING_] Mean (SD) [Min, Max] | 323 [21] 53.1 (12.6) [13.0, 82.0] |  | 122 [0] 53.3 (12.9) [21.0, 79.0] | 19 [0] 31.9 (4.8) [21.0, 38.0] | 60 [0] 50.5 (5.6) [40.0, 59.0] | 43 [0] 66.6 (4.8) [60.0, 79.0] |  | 201 [0] 52.9 (12.4) [13.0, 82.0] | 30 [0] 31.7 (6.5) [13.0, 39.0] | 108 [0] 51.4 (5.9) [40.0, 59.0] | 63 [0] 65.8 (5.2) [60.0, 82.0] |
| Sex | N [N_MISSING_] N (%) Male N (%) Female | 323 [21] 122 (37.8%) 201 (62.2%) |  | 122 [0] 122 (100.0%) 0 (0.0%) | 19 [0] 19 (100.0%) 0 (0.0%) | 60 [0] 60 (100.0%) 0 (0.0%) | 43 [0] 43 (100.0%) 0 (0.0%) |  | 201 [0] 0 (0.0%) 201 (100.0%) | 30 [0] 0 (0.0%) 30 (100.0%) | 108 [0] 0 (0.0%) 108 (100.0%) | 63 [0] 0 (0.0%) 63 (100.0%) |
| Who completed questionnaire? | N [N_MISSING_] N (%) Patient N (%) Carer N (%) Patient's Parent | 344 [0] 331 (96.2%) 6 (1.7%) 7 (2.0%) |  | 122 [0] 122 (100.0%) 0 (0.0%) 0 (0.0%) | 19 [0] 19 (100.0%) 0 (0.0%) 0 (0.0%) | 60 [0] 60 (100.0%) 0 (0.0%) 0 (0.0%) | 43 [0] 43 (100.0%) 0 (0.0%) 0 (0.0%) |  | 201 [0] 201 (100.0%) 0 (0.0%) 0 (0.0%) | 30 [0] 30 (100.0%) 0 (0.0%) 0 (0.0%) | 108 [0] 108 (100.0%) 0 (0.0%) 0 (0.0%) | 63 [0] 63 (100.0%) 0 (0.0%) 0 (0.0%) |
|  | | | | | | | | | | | | |

| **Table 3.1.2:** Age, sex and who completed the questionnaire. Sex-specific figures exclude subjects with no Age or Sex information. Subjects reporting their condition as 'Rheumatoid Arthritis'. | | | | | | | | | | | | |
| --- | --- | --- | --- | --- | --- | --- | --- | --- | --- | --- | --- | --- |
|  |  | All |  | Males | | | |  | Females | | | |
|  |  |  |  | All | < 40 | 40-59 | ≥ 60 |  | All | < 40 | 40-59 | ≥ 60 |
| Age (years) | N [N_MISSING_] Mean (SD) [Min, Max] | 189 [2] 56.0 (12.5) [20.0, 79.0] |  | 55 [0] 59.6 (12.0) [21.0, 79.0] | 3 [0] 27.3 (8.5) [21.0, 37.0] | 19 [0] 51.7 (6.4) [40.0, 59.0] | 33 [0] 67.0 (5.0) [60.0, 79.0] |  | 134 [0] 54.6 (12.4) [20.0, 77.0] | 16 [0] 30.8 (5.9) [20.0, 39.0] | 68 [0] 51.7 (5.9) [40.0, 59.0] | 50 [0] 66.1 (5.0) [60.0, 77.0] |
| Sex | N [N_MISSING_] N (%) Male N (%) Female | 189 [2] 55 (29.1%) 134 (70.9%) |  | 55 [0] 55 (100.0%) 0 (0.0%) | 3 [0] 3 (100.0%) 0 (0.0%) | 19 [0] 19 (100.0%) 0 (0.0%) | 33 [0] 33 (100.0%) 0 (0.0%) |  | 134 [0] 0 (0.0%) 134 (100.0%) | 16 [0] 0 (0.0%) 16 (100.0%) | 68 [0] 0 (0.0%) 68 (100.0%) | 50 [0] 0 (0.0%) 50 (100.0%) |
| Who completed questionnaire? | N [N_MISSING_] N (%) Patient N (%) Carer N (%) Patient's Parent | 191 [0] 189 (99.0%) 2 (1.0%) 0 (0.0%) |  | 55 [0] 55 (100.0%) 0 (0.0%) 0 (0.0%) | 3 [0] 3 (100.0%) 0 (0.0%) 0 (0.0%) | 19 [0] 19 (100.0%) 0 (0.0%) 0 (0.0%) | 33 [0] 33 (100.0%) 0 (0.0%) 0 (0.0%) |  | 134 [0] 134 (100.0%) 0 (0.0%) 0 (0.0%) | 16 [0] 16 (100.0%) 0 (0.0%) 0 (0.0%) | 68 [0] 68 (100.0%) 0 (0.0%) 0 (0.0%) | 50 [0] 50 (100.0%) 0 (0.0%) 0 (0.0%) |
|  | | | | | | | | | | | | |

| **Table 3.1.3:** Age, sex and who completed the questionnaire. Sex-specific figures exclude subjects with no Age or Sex information. Subjects reporting their condition as 'Psoriasis'. | | | | | | | | | | | | |
| --- | --- | --- | --- | --- | --- | --- | --- | --- | --- | --- | --- | --- |
|  |  | All |  | Males | | | |  | Females | | | |
|  |  |  |  | All | < 40 | 40-59 | ≥ 60 |  | All | < 40 | 40-59 | ≥ 60 |
| Age (years) | N [N_MISSING_] Mean (SD) [Min, Max] | 33 [2] 49.8 (11.0) [27.0, 82.0] |  | 15 [0] 45.3 (9.8) [27.0, 60.0] | 4 [0] 31.2 (3.3) [27.0, 35.0] | 10 [0] 49.4 (4.1) [43.0, 55.0] | 1 [0] 60.0 (-) [60.0, 60.0] |  | 18 [0] 53.6 (10.7) [33.0, 82.0] | 1 [0] 33.0 (-) [33.0, 33.0] | 12 [0] 50.1 (5.0) [40.0, 57.0] | 5 [0] 66.2 (9.0) [61.0, 82.0] |
| Sex | N [N_MISSING_] N (%) Male N (%) Female | 33 [2] 15 (45.5%) 18 (54.5%) |  | 15 [0] 15 (100.0%) 0 (0.0%) | 4 [0] 4 (100.0%) 0 (0.0%) | 10 [0] 10 (100.0%) 0 (0.0%) | 1 [0] 1 (100.0%) 0 (0.0%) |  | 18 [0] 0 (0.0%) 18 (100.0%) | 1 [0] 0 (0.0%) 1 (100.0%) | 12 [0] 0 (0.0%) 12 (100.0%) | 5 [0] 0 (0.0%) 5 (100.0%) |
| Who completed questionnaire? | N [N_MISSING_] N (%) Patient N (%) Carer N (%) Patient's Parent | 35 [0] 33 (94.3%) 1 (2.9%) 1 (2.9%) |  | 15 [0] 15 (100.0%) 0 (0.0%) 0 (0.0%) | 4 [0] 4 (100.0%) 0 (0.0%) 0 (0.0%) | 10 [0] 10 (100.0%) 0 (0.0%) 0 (0.0%) | 1 [0] 1 (100.0%) 0 (0.0%) 0 (0.0%) |  | 18 [0] 18 (100.0%) 0 (0.0%) 0 (0.0%) | 1 [0] 1 (100.0%) 0 (0.0%) 0 (0.0%) | 12 [0] 12 (100.0%) 0 (0.0%) 0 (0.0%) | 5 [0] 5 (100.0%) 0 (0.0%) 0 (0.0%) |
|  | | | | | | | | | | | | |

| **Table 3.1.4:** Age, sex and who completed the questionnaire. Sex-specific figures exclude subjects with no Age or Sex information. Subjects reporting their condition as 'Ankylosing Spondylitis'. | | | | | | | | | | | | |
| --- | --- | --- | --- | --- | --- | --- | --- | --- | --- | --- | --- | --- |
|  |  | All |  | Males | | | |  | Females | | | |
|  |  |  |  | All | < 40 | 40-59 | ≥ 60 |  | All | < 40 | 40-59 | ≥ 60 |
| Age (years) | N [N_MISSING_] Mean (SD) [Min, Max] | 43 [0] 49.2 (12.3) [27.0, 71.0] |  | 32 [0] 49.8 (12.3) [28.0, 71.0] | 8 [0] 32.9 (2.4) [28.0, 35.0] | 17 [0] 51.1 (5.3) [40.0, 59.0] | 7 [0] 66.0 (3.6) [62.0, 71.0] |  | 11 [0] 47.5 (12.5) [27.0, 66.0] | 4 [0] 34.5 (5.3) [27.0, 39.0] | 5 [0] 51.8 (7.3) [43.0, 59.0] | 2 [0] 63.0 (4.2) [60.0, 66.0] |
| Sex | N [N_MISSING_] N (%) Male N (%) Female | 43 [0] 32 (74.4%) 11 (25.6%) |  | 32 [0] 32 (100.0%) 0 (0.0%) | 8 [0] 8 (100.0%) 0 (0.0%) | 17 [0] 17 (100.0%) 0 (0.0%) | 7 [0] 7 (100.0%) 0 (0.0%) |  | 11 [0] 0 (0.0%) 11 (100.0%) | 4 [0] 0 (0.0%) 4 (100.0%) | 5 [0] 0 (0.0%) 5 (100.0%) | 2 [0] 0 (0.0%) 2 (100.0%) |
| Who completed questionnaire? | N [N_MISSING_] N (%) Patient N (%) Carer N (%) Patient's Parent | 43 [0] 43 (100.0%) 0 (0.0%) 0 (0.0%) |  | 32 [0] 32 (100.0%) 0 (0.0%) 0 (0.0%) | 8 [0] 8 (100.0%) 0 (0.0%) 0 (0.0%) | 17 [0] 17 (100.0%) 0 (0.0%) 0 (0.0%) | 7 [0] 7 (100.0%) 0 (0.0%) 0 (0.0%) |  | 11 [0] 11 (100.0%) 0 (0.0%) 0 (0.0%) | 4 [0] 4 (100.0%) 0 (0.0%) 0 (0.0%) | 5 [0] 5 (100.0%) 0 (0.0%) 0 (0.0%) | 2 [0] 2 (100.0%) 0 (0.0%) 0 (0.0%) |
|  | | | | | | | | | | | | |

| **Table 3.1.5:** Age, sex and who completed the questionnaire. Sex-specific figures exclude subjects with no Age or Sex information. Subjects reporting their condition as 'Psoriatic Arthritis'. | | | | | | | | | | | | |
| --- | --- | --- | --- | --- | --- | --- | --- | --- | --- | --- | --- | --- |
|  |  | All |  | Males | | | |  | Females | | | |
|  |  |  |  | All | < 40 | 40-59 | ≥ 60 |  | All | < 40 | 40-59 | ≥ 60 |
| Age (years) | N [N_MISSING_] Mean (SD) [Min, Max] | 41 [3] 50.5 (9.5) [34.0, 68.0] |  | 13 [0] 48.0 (8.5) [35.0, 61.0] | 3 [0] 37.0 (1.7) [35.0, 38.0] | 9 [0] 50.2 (5.9) [42.0, 59.0] | 1 [0] 61.0 (-) [61.0, 61.0] |  | 28 [0] 51.7 (9.8) [34.0, 68.0] | 5 [0] 35.6 (1.1) [34.0, 37.0] | 18 [0] 52.8 (5.5) [41.0, 59.0] | 5 [0] 63.8 (2.7) [62.0, 68.0] |
| Sex | N [N_MISSING_] N (%) Male N (%) Female | 41 [3] 13 (31.7%) 28 (68.3%) |  | 13 [0] 13 (100.0%) 0 (0.0%) | 3 [0] 3 (100.0%) 0 (0.0%) | 9 [0] 9 (100.0%) 0 (0.0%) | 1 [0] 1 (100.0%) 0 (0.0%) |  | 28 [0] 0 (0.0%) 28 (100.0%) | 5 [0] 0 (0.0%) 5 (100.0%) | 18 [0] 0 (0.0%) 18 (100.0%) | 5 [0] 0 (0.0%) 5 (100.0%) |
| Who completed questionnaire? | N [N_MISSING_] N (%) Patient N (%) Carer N (%) Patient's Parent | 44 [0] 41 (93.2%) 2 (4.5%) 1 (2.3%) |  | 13 [0] 13 (100.0%) 0 (0.0%) 0 (0.0%) | 3 [0] 3 (100.0%) 0 (0.0%) 0 (0.0%) | 9 [0] 9 (100.0%) 0 (0.0%) 0 (0.0%) | 1 [0] 1 (100.0%) 0 (0.0%) 0 (0.0%) |  | 28 [0] 28 (100.0%) 0 (0.0%) 0 (0.0%) | 5 [0] 5 (100.0%) 0 (0.0%) 0 (0.0%) | 18 [0] 18 (100.0%) 0 (0.0%) 0 (0.0%) | 5 [0] 5 (100.0%) 0 (0.0%) 0 (0.0%) |
| . | | | | | | | | | | | | |

| **Table 3.2.1:** Age, sex and who completed the questionnaire (only for those completing questionnaires online). Sex-specific figures exclude subjects with no Age or Sex information. | | | | | | | | | | | | |
| --- | --- | --- | --- | --- | --- | --- | --- | --- | --- | --- | --- | --- |
|  |  | All |  | Males | | | |  | Females | | | |
|  |  |  |  | All | < 40 | 40-59 | ≥ 60 |  | All | < 40 | 40-59 | ≥ 60 |
| Age (years) | N [N_MISSING_] Mean (SD) [Min, Max] | 269 [21] 51.4 (12.1) [13.0, 77.0] |  | 103 [0] 52.1 (12.4) [21.0, 72.0] | 18 [0] 31.7 (4.9) [21.0, 38.0] | 52 [0] 50.7 (5.7) [40.0, 59.0] | 33 [0] 65.3 (3.6) [60.0, 72.0] |  | 166 [0] 50.9 (11.9) [13.0, 77.0] | 29 [0] 31.6 (6.5) [13.0, 39.0] | 97 [0] 51.2 (5.9) [40.0, 59.0] | 40 [0] 64.3 (3.8) [60.0, 77.0] |
| Sex | N [N_MISSING_] N (%) Male N (%) Female | 269 [21] 103 (38.3%) 166 (61.7%) |  | 103 [0] 103 (100.0%) 0 (0.0%) | 18 [0] 18 (100.0%) 0 (0.0%) | 52 [0] 52 (100.0%) 0 (0.0%) | 33 [0] 33 (100.0%) 0 (0.0%) |  | 166 [0] 0 (0.0%) 166 (100.0%) | 29 [0] 0 (0.0%) 29 (100.0%) | 97 [0] 0 (0.0%) 97 (100.0%) | 40 [0] 0 (0.0%) 40 (100.0%) |
| Who completed questionnaire? | N [N_MISSING_] N (%) Patient N (%) Carer N (%) Patient's Parent | 290 [0] 277 (95.5%) 6 (2.1%) 7 (2.4%) |  | 103 [0] 103 (100.0%) 0 (0.0%) 0 (0.0%) | 18 [0] 18 (100.0%) 0 (0.0%) 0 (0.0%) | 52 [0] 52 (100.0%) 0 (0.0%) 0 (0.0%) | 33 [0] 33 (100.0%) 0 (0.0%) 0 (0.0%) |  | 166 [0] 166 (100.0%) 0 (0.0%) 0 (0.0%) | 29 [0] 29 (100.0%) 0 (0.0%) 0 (0.0%) | 97 [0] 97 (100.0%) 0 (0.0%) 0 (0.0%) | 40 [0] 40 (100.0%) 0 (0.0%) 0 (0.0%) |
| . | | | | | | | | | | | | |

| **Table 3.2.2:** Age, sex and who completed the questionnaire (only for those completing questionnaires online). Sex-specific figures exclude subjects with no Age or Sex information. Subjects reporting their condition as 'Rheumatoid Arthritis'. | | | | | | | | | | | | |
| --- | --- | --- | --- | --- | --- | --- | --- | --- | --- | --- | --- | --- |
|  |  | All |  | Males | | | |  | Females | | | |
|  |  |  |  | All | < 40 | 40-59 | ≥ 60 |  | All | < 40 | 40-59 | ≥ 60 |
| Age (years) | N [N_MISSING_] Mean (SD) [Min, Max] | 150 [2] 53.9 (12.1) [20.0, 77.0] |  | 42 [0] 57.5 (11.9) [21.0, 72.0] | 3 [0] 27.3 (8.5) [21.0, 37.0] | 16 [0] 51.7 (6.9) [40.0, 59.0] | 23 [0] 65.4 (3.6) [60.0, 72.0] |  | 108 [0] 52.5 (11.9) [20.0, 77.0] | 15 [0] 30.5 (6.0) [20.0, 39.0] | 62 [0] 51.6 (5.9) [41.0, 59.0] | 31 [0] 64.8 (4.1) [60.0, 77.0] |
| Sex | N [N_MISSING_] N (%) Male N (%) Female | 150 [2] 42 (28.0%) 108 (72.0%) |  | 42 [0] 42 (100.0%) 0 (0.0%) | 3 [0] 3 (100.0%) 0 (0.0%) | 16 [0] 16 (100.0%) 0 (0.0%) | 23 [0] 23 (100.0%) 0 (0.0%) |  | 108 [0] 0 (0.0%) 108 (100.0%) | 15 [0] 0 (0.0%) 15 (100.0%) | 62 [0] 0 (0.0%) 62 (100.0%) | 31 [0] 0 (0.0%) 31 (100.0%) |
| Who completed questionnaire? | N [N_MISSING_] N (%) Patient N (%) Carer N (%) Patient's Parent | 152 [0] 150 (98.7%) 2 (1.3%) 0 (0.0%) |  | 42 [0] 42 (100.0%) 0 (0.0%) 0 (0.0%) | 3 [0] 3 (100.0%) 0 (0.0%) 0 (0.0%) | 16 [0] 16 (100.0%) 0 (0.0%) 0 (0.0%) | 23 [0] 23 (100.0%) 0 (0.0%) 0 (0.0%) |  | 108 [0] 108 (100.0%) 0 (0.0%) 0 (0.0%) | 15 [0] 15 (100.0%) 0 (0.0%) 0 (0.0%) | 62 [0] 62 (100.0%) 0 (0.0%) 0 (0.0%) | 31 [0] 31 (100.0%) 0 (0.0%) 0 (0.0%) |
|  | | | | | | | | | | | | |

| **Table 3.2.3:** Age, sex and who completed the questionnaire (only for those completing questionnaires online). Sex-specific figures exclude subjects with no Age or Sex information. Subjects reporting their condition as 'Psoriasis'. | | | | | | | | | | | | |
| --- | --- | --- | --- | --- | --- | --- | --- | --- | --- | --- | --- | --- |
|  |  | All |  | Males | | | |  | Females | | | |
|  |  |  |  | All | < 40 | 40-59 | ≥ 60 |  | All | < 40 | 40-59 | ≥ 60 |
| Age (years) | N [N_MISSING_] Mean (SD) [Min, Max] | 29 [2] 48.2 (9.7) [27.0, 65.0] |  | 14 [0] 45.0 (10.2) [27.0, 60.0] | 4 [0] 31.2 (3.3) [27.0, 35.0] | 9 [0] 49.4 (4.3) [43.0, 55.0] | 1 [0] 60.0 (-) [60.0, 60.0] |  | 15 [0] 51.3 (8.5) [33.0, 65.0] | 1 [0] 33.0 (-) [33.0, 33.0] | 11 [0] 49.9 (5.2) [40.0, 57.0] | 3 [0] 62.3 (2.3) [61.0, 65.0] |
| Sex | N [N_MISSING_] N (%) Male N (%) Female | 29 [2] 14 (48.3%) 15 (51.7%) |  | 14 [0] 14 (100.0%) 0 (0.0%) | 4 [0] 4 (100.0%) 0 (0.0%) | 9 [0] 9 (100.0%) 0 (0.0%) | 1 [0] 1 (100.0%) 0 (0.0%) |  | 15 [0] 0 (0.0%) 15 (100.0%) | 1 [0] 0 (0.0%) 1 (100.0%) | 11 [0] 0 (0.0%) 11 (100.0%) | 3 [0] 0 (0.0%) 3 (100.0%) |
| Who completed questionnaire? | N [N_MISSING_] N (%) Patient N (%) Carer N (%) Patient's Parent | 31 [0] 29 (93.5%) 1 (3.2%) 1 (3.2%) |  | 14 [0] 14 (100.0%) 0 (0.0%) 0 (0.0%) | 4 [0] 4 (100.0%) 0 (0.0%) 0 (0.0%) | 9 [0] 9 (100.0%) 0 (0.0%) 0 (0.0%) | 1 [0] 1 (100.0%) 0 (0.0%) 0 (0.0%) |  | 15 [0] 15 (100.0%) 0 (0.0%) 0 (0.0%) | 1 [0] 1 (100.0%) 0 (0.0%) 0 (0.0%) | 11 [0] 11 (100.0%) 0 (0.0%) 0 (0.0%) | 3 [0] 3 (100.0%) 0 (0.0%) 0 (0.0%) |
|  | | | | | | | | | | | | |

| **Table 3.2.4:** Age, sex and who completed the questionnaire (only for those completing questionnaires online). Sex-specific figures exclude subjects with no Age or Sex information. Subjects reporting their condition as 'Ankylosing Spondylitis'. | | | | | | | | | | | | |
| --- | --- | --- | --- | --- | --- | --- | --- | --- | --- | --- | --- | --- |
|  |  | All |  | Males | | | |  | Females | | | |
|  |  |  |  | All | < 40 | 40-59 | ≥ 60 |  | All | < 40 | 40-59 | ≥ 60 |
| Age (years) | N [N_MISSING_] Mean (SD) [Min, Max] | 40 [0] 49.1 (12.5) [27.0, 71.0] |  | 30 [0] 49.9 (12.6) [28.0, 71.0] | 8 [0] 32.9 (2.4) [28.0, 35.0] | 15 [0] 51.5 (4.5) [42.0, 59.0] | 7 [0] 66.0 (3.6) [62.0, 71.0] |  | 10 [0] 46.6 (12.8) [27.0, 66.0] | 4 [0] 34.5 (5.3) [27.0, 39.0] | 4 [0] 50.5 (7.7) [43.0, 59.0] | 2 [0] 63.0 (4.2) [60.0, 66.0] |
| Sex | N [N_MISSING_] N (%) Male N (%) Female | 40 [0] 30 (75.0%) 10 (25.0%) |  | 30 [0] 30 (100.0%) 0 (0.0%) | 8 [0] 8 (100.0%) 0 (0.0%) | 15 [0] 15 (100.0%) 0 (0.0%) | 7 [0] 7 (100.0%) 0 (0.0%) |  | 10 [0] 0 (0.0%) 10 (100.0%) | 4 [0] 0 (0.0%) 4 (100.0%) | 4 [0] 0 (0.0%) 4 (100.0%) | 2 [0] 0 (0.0%) 2 (100.0%) |
| Who completed questionnaire? | N [N_MISSING_] N (%) Patient N (%) Carer N (%) Patient's Parent | 40 [0] 40 (100.0%) 0 (0.0%) 0 (0.0%) |  | 30 [0] 30 (100.0%) 0 (0.0%) 0 (0.0%) | 8 [0] 8 (100.0%) 0 (0.0%) 0 (0.0%) | 15 [0] 15 (100.0%) 0 (0.0%) 0 (0.0%) | 7 [0] 7 (100.0%) 0 (0.0%) 0 (0.0%) |  | 10 [0] 10 (100.0%) 0 (0.0%) 0 (0.0%) | 4 [0] 4 (100.0%) 0 (0.0%) 0 (0.0%) | 4 [0] 4 (100.0%) 0 (0.0%) 0 (0.0%) | 2 [0] 2 (100.0%) 0 (0.0%) 0 (0.0%) |
|  | | | | | | | | | | | | |

| **Table 3.2.5:** Age, sex and who completed the questionnaire (only for those completing questionnaires online). Sex-specific figures exclude subjects with no Age or Sex information. Subjects reporting their condition as 'Psoriatic Arthritis'. | | | | | | | | | | | | |
| --- | --- | --- | --- | --- | --- | --- | --- | --- | --- | --- | --- | --- |
|  |  | All |  | Males | | | |  | Females | | | |
|  |  |  |  | All | < 40 | 40-59 | ≥ 60 |  | All | < 40 | 40-59 | ≥ 60 |
| Age (years) | N [N_MISSING_] Mean (SD) [Min, Max] | 34 [3] 50.3 (9.2) [34.0, 65.0] |  | 11 [0] 49.5 (8.1) [38.0, 61.0] | 2 [0] 38.0 (0.0) [38.0, 38.0] | 8 [0] 51.0 (5.8) [42.0, 59.0] | 1 [0] 61.0 (-) [61.0, 61.0] |  | 23 [0] 50.6 (9.8) [34.0, 65.0] | 5 [0] 35.6 (1.1) [34.0, 37.0] | 15 [0] 53.1 (5.6) [41.0, 59.0] | 3 [0] 63.0 (1.7) [62.0, 65.0] |
| Sex | N [N_MISSING_] N (%) Male N (%) Female | 34 [3] 11 (32.4%) 23 (67.6%) |  | 11 [0] 11 (100.0%) 0 (0.0%) | 2 [0] 2 (100.0%) 0 (0.0%) | 8 [0] 8 (100.0%) 0 (0.0%) | 1 [0] 1 (100.0%) 0 (0.0%) |  | 23 [0] 0 (0.0%) 23 (100.0%) | 5 [0] 0 (0.0%) 5 (100.0%) | 15 [0] 0 (0.0%) 15 (100.0%) | 3 [0] 0 (0.0%) 3 (100.0%) |
| Who completed questionnaire? | N [N_MISSING_] N (%) Patient N (%) Carer N (%) Patient's Parent | 37 [0] 34 (91.9%) 2 (5.4%) 1 (2.7%) |  | 11 [0] 11 (100.0%) 0 (0.0%) 0 (0.0%) | 2 [0] 2 (100.0%) 0 (0.0%) 0 (0.0%) | 8 [0] 8 (100.0%) 0 (0.0%) 0 (0.0%) | 1 [0] 1 (100.0%) 0 (0.0%) 0 (0.0%) |  | 23 [0] 23 (100.0%) 0 (0.0%) 0 (0.0%) | 5 [0] 5 (100.0%) 0 (0.0%) 0 (0.0%) | 15 [0] 15 (100.0%) 0 (0.0%) 0 (0.0%) | 3 [0] 3 (100.0%) 0 (0.0%) 0 (0.0%) |
|  | | | | | | | | | | | | |

| **Table 3.3.1:** Age, sex and who completed the questionnaire (only for those completing questionnaires by telephone). Sex-specific figures exclude subjects with no Age or Sex information. | | | | | | | | | | | | |
| --- | --- | --- | --- | --- | --- | --- | --- | --- | --- | --- | --- | --- |
|  |  | All |  | Males | | | |  | Females | | | |
|  |  |  |  | All | < 40 | 40-59 | ≥ 60 |  | All | < 40 | 40-59 | ≥ 60 |
| Age (years) | N [N_MISSING_] Mean (SD) [Min, Max] | 54 [0] 61.5 (11.6) [35.0, 82.0] |  | 19 [0] 59.8 (13.4) [35.0, 79.0] | 1 [0] 35.0 (-) [35.0, 35.0] | 8 [0] 49.2 (5.5) [40.0, 57.0] | 10 [0] 70.7 (6.1) [60.0, 79.0] |  | 35 [0] 62.4 (10.6) [35.0, 82.0] | 1 [0] 35.0 (-) [35.0, 35.0] | 11 [0] 52.5 (5.9) [40.0, 59.0] | 23 [0] 68.3 (6.2) [60.0, 82.0] |
| Sex | N [N_MISSING_] N (%) Male N (%) Female | 54 [0] 19 (35.2%) 35 (64.8%) |  | 19 [0] 19 (100.0%) 0 (0.0%) | 1 [0] 1 (100.0%) 0 (0.0%) | 8 [0] 8 (100.0%) 0 (0.0%) | 10 [0] 10 (100.0%) 0 (0.0%) |  | 35 [0] 0 (0.0%) 35 (100.0%) | 1 [0] 0 (0.0%) 1 (100.0%) | 11 [0] 0 (0.0%) 11 (100.0%) | 23 [0] 0 (0.0%) 23 (100.0%) |
| Who completed questionnaire? | N [N_MISSING_] N (%) Patient N (%) Carer N (%) Patient's Parent | 54 [0] 54 (100.0%) 0 (0.0%) 0 (0.0%) |  | 19 [0] 19 (100.0%) 0 (0.0%) 0 (0.0%) | 1 [0] 1 (100.0%) 0 (0.0%) 0 (0.0%) | 8 [0] 8 (100.0%) 0 (0.0%) 0 (0.0%) | 10 [0] 10 (100.0%) 0 (0.0%) 0 (0.0%) |  | 35 [0] 35 (100.0%) 0 (0.0%) 0 (0.0%) | 1 [0] 1 (100.0%) 0 (0.0%) 0 (0.0%) | 11 [0] 11 (100.0%) 0 (0.0%) 0 (0.0%) | 23 [0] 23 (100.0%) 0 (0.0%) 0 (0.0%) |
| . | | | | | | | | | | | | |

| **Table 3.3.2:** Age, sex and who completed the questionnaire (only for those completing questionnaires by telephone). Sex-specific figures exclude subjects with no Age or Sex information. Subjects reporting their condition as 'Rheumatoid Arthritis'. | | | | | | | | | | | | |
| --- | --- | --- | --- | --- | --- | --- | --- | --- | --- | --- | --- | --- |
|  |  | All |  | Males | | | |  | Females | | | |
|  |  |  |  | All | < 40 | 40-59 | ≥ 60 |  | All | < 40 | 40-59 | ≥ 60 |
| Age (years) | N [N_MISSING_] Mean (SD) [Min, Max] | 39 [0] 64.4 (10.3) [35.0, 79.0] |  | 13 [0] 66.4 (9.8) [49.0, 79.0] | 0 [-] - (-) [-, -] | 3 [0] 52.0 (2.6) [49.0, 54.0] | 10 [0] 70.7 (6.1) [60.0, 79.0] |  | 26 [0] 63.4 (10.6) [35.0, 77.0] | 1 [0] 35.0 (-) [35.0, 35.0] | 6 [0] 52.5 (7.0) [40.0, 59.0] | 19 [0] 68.3 (5.6) [60.0, 77.0] |
| Sex | N [N_MISSING_] N (%) Male N (%) Female | 39 [0] 13 (33.3%) 26 (66.7%) |  | 13 [0] 13 (100.0%) 0 (0.0%) | 0 [0] 0 (-%) 0 (-%) | 3 [0] 3 (100.0%) 0 (0.0%) | 10 [0] 10 (100.0%) 0 (0.0%) |  | 26 [0] 0 (0.0%) 26 (100.0%) | 1 [0] 0 (0.0%) 1 (100.0%) | 6 [0] 0 (0.0%) 6 (100.0%) | 19 [0] 0 (0.0%) 19 (100.0%) |
| Who completed questionnaire? | N [N_MISSING_] N (%) Patient N (%) Carer N (%) Patient's Parent | 39 [0] 39 (100.0%) 0 (0.0%) 0 (0.0%) |  | 13 [0] 13 (100.0%) 0 (0.0%) 0 (0.0%) | 0 [0] 0 (-%) 0 (-%) 0 (-%) | 3 [0] 3 (100.0%) 0 (0.0%) 0 (0.0%) | 10 [0] 10 (100.0%) 0 (0.0%) 0 (0.0%) |  | 26 [0] 26 (100.0%) 0 (0.0%) 0 (0.0%) | 1 [0] 1 (100.0%) 0 (0.0%) 0 (0.0%) | 6 [0] 6 (100.0%) 0 (0.0%) 0 (0.0%) | 19 [0] 19 (100.0%) 0 (0.0%) 0 (0.0%) |
| . | | | | | | | | | | | | |

| **Table 3.3.3:** Age, sex and who completed the questionnaire (only for those completing questionnaires by telephone). Sex-specific figures exclude subjects with no Age or Sex information. Subjects reporting their condition as 'Psoriasis'. | | | | | | | | | | | | |
| --- | --- | --- | --- | --- | --- | --- | --- | --- | --- | --- | --- | --- |
|  |  | All |  | Males | | | |  | Females | | | |
|  |  |  |  | All | < 40 | 40-59 | ≥ 60 |  | All | < 40 | 40-59 | ≥ 60 |
| Age (years) | N [N_MISSING_] Mean (SD) [Min, Max] | 4 [0] 61.2 (14.9) [49.0, 82.0] |  | 1 [0] 49.0 (-) [49.0, 49.0] | 0 [-] - (-) [-, -] | 1 [0] 49.0 (-) [49.0, 49.0] | 0 [-] - (-) [-, -] |  | 3 [0] 65.3 (15.3) [52.0, 82.0] | 0 [-] - (-) [-, -] | 1 [0] 52.0 (-) [52.0, 52.0] | 2 [0] 72.0 (14.1) [62.0, 82.0] |
| Sex | N [N_MISSING_] N (%) Male N (%) Female | 4 [0] 1 (25.0%) 3 (75.0%) |  | 1 [0] 1 (100.0%) 0 (0.0%) | 0 [0] 0 (-%) 0 (-%) | 1 [0] 1 (100.0%) 0 (0.0%) | 0 [0] 0 (-%) 0 (-%) |  | 3 [0] 0 (0.0%) 3 (100.0%) | 0 [0] 0 (-%) 0 (-%) | 1 [0] 0 (0.0%) 1 (100.0%) | 2 [0] 0 (0.0%) 2 (100.0%) |
| Who completed questionnaire? | N [N_MISSING_] N (%) Patient N (%) Carer N (%) Patient's Parent | 4 [0] 4 (100.0%) 0 (0.0%) 0 (0.0%) |  | 1 [0] 1 (100.0%) 0 (0.0%) 0 (0.0%) | 0 [0] 0 (-%) 0 (-%) 0 (-%) | 1 [0] 1 (100.0%) 0 (0.0%) 0 (0.0%) | 0 [0] 0 (-%) 0 (-%) 0 (-%) |  | 3 [0] 3 (100.0%) 0 (0.0%) 0 (0.0%) | 0 [0] 0 (-%) 0 (-%) 0 (-%) | 1 [0] 1 (100.0%) 0 (0.0%) 0 (0.0%) | 2 [0] 2 (100.0%) 0 (0.0%) 0 (0.0%) |
|  | | | | | | | | | | | | |

| **Table 3.3.4:** Age, sex and who completed the questionnaire (only for those completing questionnaires by telephone). Sex-specific figures exclude subjects with no Age or Sex information. Subjects reporting their condition as 'Ankylosing Spondylitis'. | | | | | | | | | | | | |
| --- | --- | --- | --- | --- | --- | --- | --- | --- | --- | --- | --- | --- |
|  |  | All |  | Males | | | |  | Females | | | |
|  |  |  |  | All | < 40 | 40-59 | ≥ 60 |  | All | < 40 | 40-59 | ≥ 60 |
| Age (years) | N [N_MISSING_] Mean (SD) [Min, Max] | 3 [0] 51.3 (9.8) [40.0, 57.0] |  | 2 [0] 48.5 (12.0) [40.0, 57.0] | 0 [-] - (-) [-, -] | 2 [0] 48.5 (12.0) [40.0, 57.0] | 0 [-] - (-) [-, -] |  | 1 [0] 57.0 (-) [57.0, 57.0] | 0 [-] - (-) [-, -] | 1 [0] 57.0 (-) [57.0, 57.0] | 0 [-] - (-) [-, -] |
| Sex | N [N_MISSING_] N (%) Male N (%) Female | 3 [0] 2 (66.7%) 1 (33.3%) |  | 2 [0] 2 (100.0%) 0 (0.0%) | 0 [0] 0 (-%) 0 (-%) | 2 [0] 2 (100.0%) 0 (0.0%) | 0 [0] 0 (-%) 0 (-%) |  | 1 [0] 0 (0.0%) 1 (100.0%) | 0 [0] 0 (-%) 0 (-%) | 1 [0] 0 (0.0%) 1 (100.0%) | 0 [0] 0 (-%) 0 (-%) |
| Who completed questionnaire? | N [N_MISSING_] N (%) Patient N (%) Carer N (%) Patient's Parent | 3 [0] 3 (100.0%) 0 (0.0%) 0 (0.0%) |  | 2 [0] 2 (100.0%) 0 (0.0%) 0 (0.0%) | 0 [0] 0 (-%) 0 (-%) 0 (-%) | 2 [0] 2 (100.0%) 0 (0.0%) 0 (0.0%) | 0 [0] 0 (-%) 0 (-%) 0 (-%) |  | 1 [0] 1 (100.0%) 0 (0.0%) 0 (0.0%) | 0 [0] 0 (-%) 0 (-%) 0 (-%) | 1 [0] 1 (100.0%) 0 (0.0%) 0 (0.0%) | 0 [0] 0 (-%) 0 (-%) 0 (-%) |
|  | | | | | | | | | | | | |

| **Table 3.3.5:** Age, sex and who completed the questionnaire (only for those completing questionnaires by telephone). Sex-specific figures exclude subjects with no Age or Sex information. Subjects reporting their condition as 'Psoriatic Arthritis'. | | | | | | | | | | | | |
| --- | --- | --- | --- | --- | --- | --- | --- | --- | --- | --- | --- | --- |
|  |  | All |  | Males | | | |  | Females | | | |
|  |  |  |  | All | < 40 | 40-59 | ≥ 60 |  | All | < 40 | 40-59 | ≥ 60 |
| Age (years) | N [N_MISSING_] Mean (SD) [Min, Max] | 7 [0] 51.9 (11.4) [35.0, 68.0] |  | 2 [0] 39.5 (6.4) [35.0, 44.0] | 1 [0] 35.0 (-) [35.0, 35.0] | 1 [0] 44.0 (-) [44.0, 44.0] | 0 [-] - (-) [-, -] |  | 5 [0] 56.8 (8.9) [46.0, 68.0] | 0 [-] - (-) [-, -] | 3 [0] 51.3 (6.1) [46.0, 58.0] | 2 [0] 65.0 (4.2) [62.0, 68.0] |
| Sex | N [N_MISSING_] N (%) Male N (%) Female | 7 [0] 2 (28.6%) 5 (71.4%) |  | 2 [0] 2 (100.0%) 0 (0.0%) | 1 [0] 1 (100.0%) 0 (0.0%) | 1 [0] 1 (100.0%) 0 (0.0%) | 0 [0] 0 (-%) 0 (-%) |  | 5 [0] 0 (0.0%) 5 (100.0%) | 0 [0] 0 (-%) 0 (-%) | 3 [0] 0 (0.0%) 3 (100.0%) | 2 [0] 0 (0.0%) 2 (100.0%) |
| Who completed questionnaire? | N [N_MISSING_] N (%) Patient N (%) Carer N (%) Patient's Parent | 7 [0] 7 (100.0%) 0 (0.0%) 0 (0.0%) |  | 2 [0] 2 (100.0%) 0 (0.0%) 0 (0.0%) | 1 [0] 1 (100.0%) 0 (0.0%) 0 (0.0%) | 1 [0] 1 (100.0%) 0 (0.0%) 0 (0.0%) | 0 [0] 0 (-%) 0 (-%) 0 (-%) |  | 5 [0] 5 (100.0%) 0 (0.0%) 0 (0.0%) | 0 [0] 0 (-%) 0 (-%) 0 (-%) | 3 [0] 3 (100.0%) 0 (0.0%) 0 (0.0%) | 2 [0] 2 (100.0%) 0 (0.0%) 0 (0.0%) |
|  | | | | | | | | | | | | |

| **Table 5.1.1:** Current medications at baseline. Sex-specific figures exclude subjects with no Age or Sex information. | | | | | | | | | | | | |
| --- | --- | --- | --- | --- | --- | --- | --- | --- | --- | --- | --- | --- |
|  |  | All |  | Males | | | |  | Females | | | |
|  |  |  |  | All | < 40 | 40-59 | ≥ 60 |  | All | < 40 | 40-59 | ≥ 60 |
| Currently taking Sulfasalazine? | N [N_MISSING_] N (%) No N (%) Yes | 331 [13] 278 (84.0%) 53 (16.0%) |  | 120 [2] 103 (85.8%) 17 (14.2%) | 19 [0] 14 (73.7%) 5 (26.3%) | 58 [2] 51 (87.9%) 7 (12.1%) | 43 [0] 38 (88.4%) 5 (11.6%) |  | 198 [3] 162 (81.8%) 36 (18.2%) | 29 [1] 23 (79.3%) 6 (20.7%) | 106 [2] 87 (82.1%) 19 (17.9%) | 63 [0] 52 (82.5%) 11 (17.5%) |
| Currently taking Leflunomide? | N [N_MISSING_] N (%) No N (%) Yes | 331 [13] 313 (94.6%) 18 (5.4%) |  | 120 [2] 115 (95.8%) 5 (4.2%) | 19 [0] 17 (89.5%) 2 (10.5%) | 58 [2] 56 (96.6%) 2 (3.4%) | 43 [0] 42 (97.7%) 1 (2.3%) |  | 198 [3] 186 (93.9%) 12 (6.1%) | 29 [1] 28 (96.6%) 1 (3.4%) | 106 [2] 100 (94.3%) 6 (5.7%) | 63 [0] 58 (92.1%) 5 (7.9%) |
| Currently taking Prednisolone? | N [N_MISSING_] N (%) No N (%) Yes | 331 [13] 262 (79.2%) 69 (20.8%) |  | 120 [2] 92 (76.7%) 28 (23.3%) | 19 [0] 16 (84.2%) 3 (15.8%) | 58 [2] 51 (87.9%) 7 (12.1%) | 43 [0] 25 (58.1%) 18 (41.9%) |  | 198 [3] 159 (80.3%) 39 (19.7%) | 29 [1] 23 (79.3%) 6 (20.7%) | 106 [2] 87 (82.1%) 19 (17.9%) | 63 [0] 49 (77.8%) 14 (22.2%) |
| Currently taking Cyclosporin? | N [N_MISSING_] N (%) No N (%) Yes | 331 [13] 326 (98.5%) 5 (1.5%) |  | 120 [2] 118 (98.3%) 2 (1.7%) | 19 [0] 18 (94.7%) 1 (5.3%) | 58 [2] 58 (100.0%) 0 (0.0%) | 43 [0] 42 (97.7%) 1 (2.3%) |  | 198 [3] 196 (99.0%) 2 (1.0%) | 29 [1] 29 (100.0%) 0 (0.0%) | 106 [2] 104 (98.1%) 2 (1.9%) | 63 [0] 63 (100.0%) 0 (0.0%) |
| Currently taking Acitretin? | N [N_MISSING_] N (%) No N (%) Yes | 331 [13] 326 (98.5%) 5 (1.5%) |  | 120 [2] 117 (97.5%) 3 (2.5%) | 19 [0] 18 (94.7%) 1 (5.3%) | 58 [2] 56 (96.6%) 2 (3.4%) | 43 [0] 43 (100.0%) 0 (0.0%) |  | 198 [3] 196 (99.0%) 2 (1.0%) | 29 [1] 29 (100.0%) 0 (0.0%) | 106 [2] 104 (98.1%) 2 (1.9%) | 63 [0] 63 (100.0%) 0 (0.0%) |
| Currently receiving Phototherapy? | N [N_MISSING_] N (%) No N (%) Yes | 35 [309] 35 (100.0%) 0 (0.0%) |  | 15 [107] 15 (100.0%) 0 (0.0%) | 4 [15] 4 (100.0%) 0 (0.0%) | 10 [50] 10 (100.0%) 0 (0.0%) | 1 [42] 1 (100.0%) 0 (0.0%) |  | 18 [183] 18 (100.0%) 0 (0.0%) | 1 [29] 1 (100.0%) 0 (0.0%) | 12 [96] 12 (100.0%) 0 (0.0%) | 5 [58] 5 (100.0%) 0 (0.0%) |
| Currently using any creams for psoriasis? | N [N_MISSING_] N (%) No N (%) Yes | 35 [309] 10 (28.6%) 25 (71.4%) |  | 15 [107] 4 (26.7%) 11 (73.3%) | 4 [15] 2 (50.0%) 2 (50.0%) | 10 [50] 2 (20.0%) 8 (80.0%) | 1 [42] 0 (0.0%) 1 (100.0%) |  | 18 [183] 5 (27.8%) 13 (72.2%) | 1 [29] 0 (0.0%) 1 (100.0%) | 12 [96] 3 (25.0%) 9 (75.0%) | 5 [58] 2 (40.0%) 3 (60.0%) |
|  | | | | | | | | | | | | |

| **Table 5.1.2:** Current medications at baseline. Sex-specific figures exclude subjects with no Age or Sex information. Subjects reporting their condition as 'Rheumatoid Arthritis'. | | | | | | | | | | | | |
| --- | --- | --- | --- | --- | --- | --- | --- | --- | --- | --- | --- | --- |
|  |  | All |  | Males | | | |  | Females | | | |
|  |  |  |  | All | < 40 | 40-59 | ≥ 60 |  | All | < 40 | 40-59 | ≥ 60 |
| Currently taking Sulfasalazine? | N [N_MISSING_] N (%) No N (%) Yes | 190 [1] 149 (78.4%) 41 (21.6%) |  | 55 [0] 45 (81.8%) 10 (18.2%) | 3 [0] 1 (33.3%) 2 (66.7%) | 19 [0] 16 (84.2%) 3 (15.8%) | 33 [0] 28 (84.8%) 5 (15.2%) |  | 133 [1] 102 (76.7%) 31 (23.3%) | 16 [0] 11 (68.8%) 5 (31.2%) | 67 [1] 51 (76.1%) 16 (23.9%) | 50 [0] 40 (80.0%) 10 (20.0%) |
| Currently taking Leflunomide? | N [N_MISSING_] N (%) No N (%) Yes | 190 [1] 176 (92.6%) 14 (7.4%) |  | 55 [0] 52 (94.5%) 3 (5.5%) | 3 [0] 2 (66.7%) 1 (33.3%) | 19 [0] 18 (94.7%) 1 (5.3%) | 33 [0] 32 (97.0%) 1 (3.0%) |  | 133 [1] 123 (92.5%) 10 (7.5%) | 16 [0] 15 (93.8%) 1 (6.2%) | 67 [1] 62 (92.5%) 5 (7.5%) | 50 [0] 46 (92.0%) 4 (8.0%) |
| Currently taking Prednisolone? | N [N_MISSING_] N (%) No N (%) Yes | 190 [1] 137 (72.1%) 53 (27.9%) |  | 55 [0] 34 (61.8%) 21 (38.2%) | 3 [0] 2 (66.7%) 1 (33.3%) | 19 [0] 14 (73.7%) 5 (26.3%) | 33 [0] 18 (54.5%) 15 (45.5%) |  | 133 [1] 101 (75.9%) 32 (24.1%) | 16 [0] 11 (68.8%) 5 (31.2%) | 67 [1] 52 (77.6%) 15 (22.4%) | 50 [0] 38 (76.0%) 12 (24.0%) |
| Currently taking Cyclosporin? | N [N_MISSING_] N (%) No N (%) Yes | 190 [1] 188 (98.9%) 2 (1.1%) |  | 55 [0] 55 (100.0%) 0 (0.0%) | 3 [0] 3 (100.0%) 0 (0.0%) | 19 [0] 19 (100.0%) 0 (0.0%) | 33 [0] 33 (100.0%) 0 (0.0%) |  | 133 [1] 131 (98.5%) 2 (1.5%) | 16 [0] 16 (100.0%) 0 (0.0%) | 67 [1] 65 (97.0%) 2 (3.0%) | 50 [0] 50 (100.0%) 0 (0.0%) |
| Currently taking Acitretin? | N [N_MISSING_] N (%) No N (%) Yes | 190 [1] 187 (98.4%) 3 (1.6%) |  | 55 [0] 53 (96.4%) 2 (3.6%) | 3 [0] 3 (100.0%) 0 (0.0%) | 19 [0] 17 (89.5%) 2 (10.5%) | 33 [0] 33 (100.0%) 0 (0.0%) |  | 133 [1] 132 (99.2%) 1 (0.8%) | 16 [0] 16 (100.0%) 0 (0.0%) | 67 [1] 66 (98.5%) 1 (1.5%) | 50 [0] 50 (100.0%) 0 (0.0%) |
| Currently receiving Phototherapy? | N [N_MISSING_] N (%) No N (%) Yes | 0 [191] 0 (-%) 0 (-%) |  | 0 [55] 0 (-%) 0 (-%) | 0 [3] 0 (-%) 0 (-%) | 0 [19] 0 (-%) 0 (-%) | 0 [33] 0 (-%) 0 (-%) |  | 0 [134] 0 (-%) 0 (-%) | 0 [16] 0 (-%) 0 (-%) | 0 [68] 0 (-%) 0 (-%) | 0 [50] 0 (-%) 0 (-%) |
| Currently using any creams for psoriasis? | N [N_MISSING_] N (%) No N (%) Yes | 0 [191] 0 (-%) 0 (-%) |  | 0 [55] 0 (-%) 0 (-%) | 0 [3] 0 (-%) 0 (-%) | 0 [19] 0 (-%) 0 (-%) | 0 [33] 0 (-%) 0 (-%) |  | 0 [134] 0 (-%) 0 (-%) | 0 [16] 0 (-%) 0 (-%) | 0 [68] 0 (-%) 0 (-%) | 0 [50] 0 (-%) 0 (-%) |
| . | | | | | | | | | | | | |

| **Table 5.1.3:** Current medications at baseline. Sex-specific figures exclude subjects with no Age or Sex information. Subjects reporting their condition as 'Psoriasis'. | | | | | | | | | | | | |
| --- | --- | --- | --- | --- | --- | --- | --- | --- | --- | --- | --- | --- |
|  |  | All |  | Males | | | |  | Females | | | |
|  |  |  |  | All | < 40 | 40-59 | ≥ 60 |  | All | < 40 | 40-59 | ≥ 60 |
| Currently taking Sulfasalazine? | N [N_MISSING_] N (%) No N (%) Yes | 35 [0] 35 (100.0%) 0 (0.0%) |  | 15 [0] 15 (100.0%) 0 (0.0%) | 4 [0] 4 (100.0%) 0 (0.0%) | 10 [0] 10 (100.0%) 0 (0.0%) | 1 [0] 1 (100.0%) 0 (0.0%) |  | 18 [0] 18 (100.0%) 0 (0.0%) | 1 [0] 1 (100.0%) 0 (0.0%) | 12 [0] 12 (100.0%) 0 (0.0%) | 5 [0] 5 (100.0%) 0 (0.0%) |
| Currently taking Leflunomide? | N [N_MISSING_] N (%) No N (%) Yes | 35 [0] 35 (100.0%) 0 (0.0%) |  | 15 [0] 15 (100.0%) 0 (0.0%) | 4 [0] 4 (100.0%) 0 (0.0%) | 10 [0] 10 (100.0%) 0 (0.0%) | 1 [0] 1 (100.0%) 0 (0.0%) |  | 18 [0] 18 (100.0%) 0 (0.0%) | 1 [0] 1 (100.0%) 0 (0.0%) | 12 [0] 12 (100.0%) 0 (0.0%) | 5 [0] 5 (100.0%) 0 (0.0%) |
| Currently taking Prednisolone? | N [N_MISSING_] N (%) No N (%) Yes | 35 [0] 34 (97.1%) 1 (2.9%) |  | 15 [0] 15 (100.0%) 0 (0.0%) | 4 [0] 4 (100.0%) 0 (0.0%) | 10 [0] 10 (100.0%) 0 (0.0%) | 1 [0] 1 (100.0%) 0 (0.0%) |  | 18 [0] 17 (94.4%) 1 (5.6%) | 1 [0] 1 (100.0%) 0 (0.0%) | 12 [0] 12 (100.0%) 0 (0.0%) | 5 [0] 4 (80.0%) 1 (20.0%) |
| Currently taking Cyclosporin? | N [N_MISSING_] N (%) No N (%) Yes | 35 [0] 34 (97.1%) 1 (2.9%) |  | 15 [0] 14 (93.3%) 1 (6.7%) | 4 [0] 3 (75.0%) 1 (25.0%) | 10 [0] 10 (100.0%) 0 (0.0%) | 1 [0] 1 (100.0%) 0 (0.0%) |  | 18 [0] 18 (100.0%) 0 (0.0%) | 1 [0] 1 (100.0%) 0 (0.0%) | 12 [0] 12 (100.0%) 0 (0.0%) | 5 [0] 5 (100.0%) 0 (0.0%) |
| Currently taking Acitretin? | N [N_MISSING_] N (%) No N (%) Yes | 35 [0] 33 (94.3%) 2 (5.7%) |  | 15 [0] 14 (93.3%) 1 (6.7%) | 4 [0] 3 (75.0%) 1 (25.0%) | 10 [0] 10 (100.0%) 0 (0.0%) | 1 [0] 1 (100.0%) 0 (0.0%) |  | 18 [0] 17 (94.4%) 1 (5.6%) | 1 [0] 1 (100.0%) 0 (0.0%) | 12 [0] 11 (91.7%) 1 (8.3%) | 5 [0] 5 (100.0%) 0 (0.0%) |
| Currently receiving Phototherapy? | N [N_MISSING_] N (%) No N (%) Yes | 35 [0] 35 (100.0%) 0 (0.0%) |  | 15 [0] 15 (100.0%) 0 (0.0%) | 4 [0] 4 (100.0%) 0 (0.0%) | 10 [0] 10 (100.0%) 0 (0.0%) | 1 [0] 1 (100.0%) 0 (0.0%) |  | 18 [0] 18 (100.0%) 0 (0.0%) | 1 [0] 1 (100.0%) 0 (0.0%) | 12 [0] 12 (100.0%) 0 (0.0%) | 5 [0] 5 (100.0%) 0 (0.0%) |
| Currently using any creams for psoriasis? | N [N_MISSING_] N (%) No N (%) Yes | 35 [0] 10 (28.6%) 25 (71.4%) |  | 15 [0] 4 (26.7%) 11 (73.3%) | 4 [0] 2 (50.0%) 2 (50.0%) | 10 [0] 2 (20.0%) 8 (80.0%) | 1 [0] 0 (0.0%) 1 (100.0%) |  | 18 [0] 5 (27.8%) 13 (72.2%) | 1 [0] 0 (0.0%) 1 (100.0%) | 12 [0] 3 (25.0%) 9 (75.0%) | 5 [0] 2 (40.0%) 3 (60.0%) |
| . | | | | | | | | | | | | |

| **Table 5.1.4:** Current medications at baseline. Sex-specific figures exclude subjects with no Age or Sex information. Subjects reporting their condition as 'Ankylosing Spondylitis'. | | | | | | | | | | | | |
| --- | --- | --- | --- | --- | --- | --- | --- | --- | --- | --- | --- | --- |
|  |  | All |  | Males | | | |  | Females | | | |
|  |  |  |  | All | < 40 | 40-59 | ≥ 60 |  | All | < 40 | 40-59 | ≥ 60 |
| Currently taking Sulfasalazine? | N [N_MISSING_] N (%) No N (%) Yes | 43 [0] 39 (90.7%) 4 (9.3%) |  | 32 [0] 29 (90.6%) 3 (9.4%) | 8 [0] 7 (87.5%) 1 (12.5%) | 17 [0] 15 (88.2%) 2 (11.8%) | 7 [0] 7 (100.0%) 0 (0.0%) |  | 11 [0] 10 (90.9%) 1 (9.1%) | 4 [0] 4 (100.0%) 0 (0.0%) | 5 [0] 5 (100.0%) 0 (0.0%) | 2 [0] 1 (50.0%) 1 (50.0%) |
| Currently taking Leflunomide? | N [N_MISSING_] N (%) No N (%) Yes | 43 [0] 43 (100.0%) 0 (0.0%) |  | 32 [0] 32 (100.0%) 0 (0.0%) | 8 [0] 8 (100.0%) 0 (0.0%) | 17 [0] 17 (100.0%) 0 (0.0%) | 7 [0] 7 (100.0%) 0 (0.0%) |  | 11 [0] 11 (100.0%) 0 (0.0%) | 4 [0] 4 (100.0%) 0 (0.0%) | 5 [0] 5 (100.0%) 0 (0.0%) | 2 [0] 2 (100.0%) 0 (0.0%) |
| Currently taking Prednisolone? | N [N_MISSING_] N (%) No N (%) Yes | 43 [0] 37 (86.0%) 6 (14.0%) |  | 32 [0] 27 (84.4%) 5 (15.6%) | 8 [0] 7 (87.5%) 1 (12.5%) | 17 [0] 16 (94.1%) 1 (5.9%) | 7 [0] 4 (57.1%) 3 (42.9%) |  | 11 [0] 10 (90.9%) 1 (9.1%) | 4 [0] 4 (100.0%) 0 (0.0%) | 5 [0] 5 (100.0%) 0 (0.0%) | 2 [0] 1 (50.0%) 1 (50.0%) |
| Currently taking Cyclosporin? | N [N_MISSING_] N (%) No N (%) Yes | 43 [0] 43 (100.0%) 0 (0.0%) |  | 32 [0] 32 (100.0%) 0 (0.0%) | 8 [0] 8 (100.0%) 0 (0.0%) | 17 [0] 17 (100.0%) 0 (0.0%) | 7 [0] 7 (100.0%) 0 (0.0%) |  | 11 [0] 11 (100.0%) 0 (0.0%) | 4 [0] 4 (100.0%) 0 (0.0%) | 5 [0] 5 (100.0%) 0 (0.0%) | 2 [0] 2 (100.0%) 0 (0.0%) |
| Currently taking Acitretin? | N [N_MISSING_] N (%) No N (%) Yes | 43 [0] 43 (100.0%) 0 (0.0%) |  | 32 [0] 32 (100.0%) 0 (0.0%) | 8 [0] 8 (100.0%) 0 (0.0%) | 17 [0] 17 (100.0%) 0 (0.0%) | 7 [0] 7 (100.0%) 0 (0.0%) |  | 11 [0] 11 (100.0%) 0 (0.0%) | 4 [0] 4 (100.0%) 0 (0.0%) | 5 [0] 5 (100.0%) 0 (0.0%) | 2 [0] 2 (100.0%) 0 (0.0%) |
| Currently receiving Phototherapy? | N [N_MISSING_] N (%) No N (%) Yes | 0 [43] 0 (-%) 0 (-%) |  | 0 [32] 0 (-%) 0 (-%) | 0 [8] 0 (-%) 0 (-%) | 0 [17] 0 (-%) 0 (-%) | 0 [7] 0 (-%) 0 (-%) |  | 0 [11] 0 (-%) 0 (-%) | 0 [4] 0 (-%) 0 (-%) | 0 [5] 0 (-%) 0 (-%) | 0 [2] 0 (-%) 0 (-%) |
| Currently using any creams for psoriasis? | N [N_MISSING_] N (%) No N (%) Yes | 0 [43] 0 (-%) 0 (-%) |  | 0 [32] 0 (-%) 0 (-%) | 0 [8] 0 (-%) 0 (-%) | 0 [17] 0 (-%) 0 (-%) | 0 [7] 0 (-%) 0 (-%) |  | 0 [11] 0 (-%) 0 (-%) | 0 [4] 0 (-%) 0 (-%) | 0 [5] 0 (-%) 0 (-%) | 0 [2] 0 (-%) 0 (-%) |
| . | | | | | | | | | | | | |

| **Table 5.1.5:** Current medications at baseline. Sex-specific figures exclude subjects with no Age or Sex information. Subjects reporting their condition as 'Psoriatic Arthritis'. | | | | | | | | | | | | |
| --- | --- | --- | --- | --- | --- | --- | --- | --- | --- | --- | --- | --- |
|  |  | All |  | Males | | | |  | Females | | | |
|  |  |  |  | All | < 40 | 40-59 | ≥ 60 |  | All | < 40 | 40-59 | ≥ 60 |
| Currently taking Sulfasalazine? | N [N_MISSING_] N (%) No N (%) Yes | 44 [0] 38 (86.4%) 6 (13.6%) |  | 13 [0] 10 (76.9%) 3 (23.1%) | 3 [0] 1 (33.3%) 2 (66.7%) | 9 [0] 8 (88.9%) 1 (11.1%) | 1 [0] 1 (100.0%) 0 (0.0%) |  | 28 [0] 25 (89.3%) 3 (10.7%) | 5 [0] 5 (100.0%) 0 (0.0%) | 18 [0] 15 (83.3%) 3 (16.7%) | 5 [0] 5 (100.0%) 0 (0.0%) |
| Currently taking Leflunomide? | N [N_MISSING_] N (%) No N (%) Yes | 44 [0] 40 (90.9%) 4 (9.1%) |  | 13 [0] 11 (84.6%) 2 (15.4%) | 3 [0] 2 (66.7%) 1 (33.3%) | 9 [0] 8 (88.9%) 1 (11.1%) | 1 [0] 1 (100.0%) 0 (0.0%) |  | 28 [0] 26 (92.9%) 2 (7.1%) | 5 [0] 5 (100.0%) 0 (0.0%) | 18 [0] 17 (94.4%) 1 (5.6%) | 5 [0] 4 (80.0%) 1 (20.0%) |
| Currently taking Prednisolone? | N [N_MISSING_] N (%) No N (%) Yes | 44 [0] 39 (88.6%) 5 (11.4%) |  | 13 [0] 12 (92.3%) 1 (7.7%) | 3 [0] 3 (100.0%) 0 (0.0%) | 9 [0] 8 (88.9%) 1 (11.1%) | 1 [0] 1 (100.0%) 0 (0.0%) |  | 28 [0] 25 (89.3%) 3 (10.7%) | 5 [0] 4 (80.0%) 1 (20.0%) | 18 [0] 16 (88.9%) 2 (11.1%) | 5 [0] 5 (100.0%) 0 (0.0%) |
| Currently taking Cyclosporin? | N [N_MISSING_] N (%) No N (%) Yes | 44 [0] 43 (97.7%) 1 (2.3%) |  | 13 [0] 12 (92.3%) 1 (7.7%) | 3 [0] 3 (100.0%) 0 (0.0%) | 9 [0] 9 (100.0%) 0 (0.0%) | 1 [0] 0 (0.0%) 1 (100.0%) |  | 28 [0] 28 (100.0%) 0 (0.0%) | 5 [0] 5 (100.0%) 0 (0.0%) | 18 [0] 18 (100.0%) 0 (0.0%) | 5 [0] 5 (100.0%) 0 (0.0%) |
| Currently taking Acitretin? | N [N_MISSING_] N (%) No N (%) Yes | 44 [0] 44 (100.0%) 0 (0.0%) |  | 13 [0] 13 (100.0%) 0 (0.0%) | 3 [0] 3 (100.0%) 0 (0.0%) | 9 [0] 9 (100.0%) 0 (0.0%) | 1 [0] 1 (100.0%) 0 (0.0%) |  | 28 [0] 28 (100.0%) 0 (0.0%) | 5 [0] 5 (100.0%) 0 (0.0%) | 18 [0] 18 (100.0%) 0 (0.0%) | 5 [0] 5 (100.0%) 0 (0.0%) |
| Currently receiving Phototherapy? | N [N_MISSING_] N (%) No N (%) Yes | 0 [44] 0 (-%) 0 (-%) |  | 0 [13] 0 (-%) 0 (-%) | 0 [3] 0 (-%) 0 (-%) | 0 [9] 0 (-%) 0 (-%) | 0 [1] 0 (-%) 0 (-%) |  | 0 [28] 0 (-%) 0 (-%) | 0 [5] 0 (-%) 0 (-%) | 0 [18] 0 (-%) 0 (-%) | 0 [5] 0 (-%) 0 (-%) |
| Currently using any creams for psoriasis? | N [N_MISSING_] N (%) No N (%) Yes | 0 [44] 0 (-%) 0 (-%) |  | 0 [13] 0 (-%) 0 (-%) | 0 [3] 0 (-%) 0 (-%) | 0 [9] 0 (-%) 0 (-%) | 0 [1] 0 (-%) 0 (-%) |  | 0 [28] 0 (-%) 0 (-%) | 0 [5] 0 (-%) 0 (-%) | 0 [18] 0 (-%) 0 (-%) | 0 [5] 0 (-%) 0 (-%) |
| . | | | | | | | | | | | | |

| **Table 5.2.1:** Current medications at baseline (only for those completing questionnaires online). Sex-specific figures exclude subjects with no Age or Sex information. | | | | | | | | | | | | |
| --- | --- | --- | --- | --- | --- | --- | --- | --- | --- | --- | --- | --- |
|  |  | All |  | Males | | | |  | Females | | | |
|  |  |  |  | All | < 40 | 40-59 | ≥ 60 |  | All | < 40 | 40-59 | ≥ 60 |
| Currently taking Sulfasalazine? | N [N_MISSING_] N (%) No N (%) Yes | 277 [13] 231 (83.4%) 46 (16.6%) |  | 101 [2] 86 (85.1%) 15 (14.9%) | 18 [0] 13 (72.2%) 5 (27.8%) | 50 [2] 43 (86.0%) 7 (14.0%) | 33 [0] 30 (90.9%) 3 (9.1%) |  | 163 [3] 132 (81.0%) 31 (19.0%) | 28 [1] 23 (82.1%) 5 (17.9%) | 95 [2] 78 (82.1%) 17 (17.9%) | 40 [0] 31 (77.5%) 9 (22.5%) |
| Currently taking Leflunomide? | N [N_MISSING_] N (%) No N (%) Yes | 277 [13] 260 (93.9%) 17 (6.1%) |  | 101 [2] 97 (96.0%) 4 (4.0%) | 18 [0] 17 (94.4%) 1 (5.6%) | 50 [2] 48 (96.0%) 2 (4.0%) | 33 [0] 32 (97.0%) 1 (3.0%) |  | 163 [3] 151 (92.6%) 12 (7.4%) | 28 [1] 27 (96.4%) 1 (3.6%) | 95 [2] 89 (93.7%) 6 (6.3%) | 40 [0] 35 (87.5%) 5 (12.5%) |
| Currently taking Prednisolone? | N [N_MISSING_] N (%) No N (%) Yes | 277 [13] 222 (80.1%) 55 (19.9%) |  | 101 [2] 78 (77.2%) 23 (22.8%) | 18 [0] 15 (83.3%) 3 (16.7%) | 50 [2] 43 (86.0%) 7 (14.0%) | 33 [0] 20 (60.6%) 13 (39.4%) |  | 163 [3] 133 (81.6%) 30 (18.4%) | 28 [1] 22 (78.6%) 6 (21.4%) | 95 [2] 77 (81.1%) 18 (18.9%) | 40 [0] 34 (85.0%) 6 (15.0%) |
| Currently taking Cyclosporin? | N [N_MISSING_] N (%) No N (%) Yes | 277 [13] 272 (98.2%) 5 (1.8%) |  | 101 [2] 99 (98.0%) 2 (2.0%) | 18 [0] 17 (94.4%) 1 (5.6%) | 50 [2] 50 (100.0%) 0 (0.0%) | 33 [0] 32 (97.0%) 1 (3.0%) |  | 163 [3] 161 (98.8%) 2 (1.2%) | 28 [1] 28 (100.0%) 0 (0.0%) | 95 [2] 93 (97.9%) 2 (2.1%) | 40 [0] 40 (100.0%) 0 (0.0%) |
| Currently taking Acitretin? | N [N_MISSING_] N (%) No N (%) Yes | 277 [13] 272 (98.2%) 5 (1.8%) |  | 101 [2] 98 (97.0%) 3 (3.0%) | 18 [0] 17 (94.4%) 1 (5.6%) | 50 [2] 48 (96.0%) 2 (4.0%) | 33 [0] 33 (100.0%) 0 (0.0%) |  | 163 [3] 161 (98.8%) 2 (1.2%) | 28 [1] 28 (100.0%) 0 (0.0%) | 95 [2] 93 (97.9%) 2 (2.1%) | 40 [0] 40 (100.0%) 0 (0.0%) |
| Currently receiving Phototherapy? | N [N_MISSING_] N (%) No N (%) Yes | 31 [259] 31 (100.0%) 0 (0.0%) |  | 14 [89] 14 (100.0%) 0 (0.0%) | 4 [14] 4 (100.0%) 0 (0.0%) | 9 [43] 9 (100.0%) 0 (0.0%) | 1 [32] 1 (100.0%) 0 (0.0%) |  | 15 [151] 15 (100.0%) 0 (0.0%) | 1 [28] 1 (100.0%) 0 (0.0%) | 11 [86] 11 (100.0%) 0 (0.0%) | 3 [37] 3 (100.0%) 0 (0.0%) |
| Currently using any creams for psoriasis? | N [N_MISSING_] N (%) No N (%) Yes | 31 [259] 9 (29.0%) 22 (71.0%) |  | 14 [89] 4 (28.6%) 10 (71.4%) | 4 [14] 2 (50.0%) 2 (50.0%) | 9 [43] 2 (22.2%) 7 (77.8%) | 1 [32] 0 (0.0%) 1 (100.0%) |  | 15 [151] 4 (26.7%) 11 (73.3%) | 1 [28] 0 (0.0%) 1 (100.0%) | 11 [86] 3 (27.3%) 8 (72.7%) | 3 [37] 1 (33.3%) 2 (66.7%) |
|  | | | | | | | | | | | | |

| **Table 5.3.1:** Current medications at baseline (only for those completing questionnaires by telephone). Sex-specific figures exclude subjects with no Age or Sex information. | | | | | | | | | | | | |
| --- | --- | --- | --- | --- | --- | --- | --- | --- | --- | --- | --- | --- |
|  |  | All |  | Males | | | |  | Females | | | |
|  |  |  |  | All | < 40 | 40-59 | ≥ 60 |  | All | < 40 | 40-59 | ≥ 60 |
| Currently taking Sulfasalazine? | N [N_MISSING_] N (%) No N (%) Yes | 54 [0] 47 (87.0%) 7 (13.0%) |  | 19 [0] 17 (89.5%) 2 (10.5%) | 1 [0] 1 (100.0%) 0 (0.0%) | 8 [0] 8 (100.0%) 0 (0.0%) | 10 [0] 8 (80.0%) 2 (20.0%) |  | 35 [0] 30 (85.7%) 5 (14.3%) | 1 [0] 0 (0.0%) 1 (100.0%) | 11 [0] 9 (81.8%) 2 (18.2%) | 23 [0] 21 (91.3%) 2 (8.7%) |
| Currently taking Leflunomide? | N [N_MISSING_] N (%) No N (%) Yes | 54 [0] 53 (98.1%) 1 (1.9%) |  | 19 [0] 18 (94.7%) 1 (5.3%) | 1 [0] 0 (0.0%) 1 (100.0%) | 8 [0] 8 (100.0%) 0 (0.0%) | 10 [0] 10 (100.0%) 0 (0.0%) |  | 35 [0] 35 (100.0%) 0 (0.0%) | 1 [0] 1 (100.0%) 0 (0.0%) | 11 [0] 11 (100.0%) 0 (0.0%) | 23 [0] 23 (100.0%) 0 (0.0%) |
| Currently taking Prednisolone? | N [N_MISSING_] N (%) No N (%) Yes | 54 [0] 40 (74.1%) 14 (25.9%) |  | 19 [0] 14 (73.7%) 5 (26.3%) | 1 [0] 1 (100.0%) 0 (0.0%) | 8 [0] 8 (100.0%) 0 (0.0%) | 10 [0] 5 (50.0%) 5 (50.0%) |  | 35 [0] 26 (74.3%) 9 (25.7%) | 1 [0] 1 (100.0%) 0 (0.0%) | 11 [0] 10 (90.9%) 1 (9.1%) | 23 [0] 15 (65.2%) 8 (34.8%) |
| Currently taking Cyclosporin? | N [N_MISSING_] N (%) No N (%) Yes | 54 [0] 54 (100.0%) 0 (0.0%) |  | 19 [0] 19 (100.0%) 0 (0.0%) | 1 [0] 1 (100.0%) 0 (0.0%) | 8 [0] 8 (100.0%) 0 (0.0%) | 10 [0] 10 (100.0%) 0 (0.0%) |  | 35 [0] 35 (100.0%) 0 (0.0%) | 1 [0] 1 (100.0%) 0 (0.0%) | 11 [0] 11 (100.0%) 0 (0.0%) | 23 [0] 23 (100.0%) 0 (0.0%) |
| Currently taking Acitretin? | N [N_MISSING_] N (%) No N (%) Yes | 54 [0] 54 (100.0%) 0 (0.0%) |  | 19 [0] 19 (100.0%) 0 (0.0%) | 1 [0] 1 (100.0%) 0 (0.0%) | 8 [0] 8 (100.0%) 0 (0.0%) | 10 [0] 10 (100.0%) 0 (0.0%) |  | 35 [0] 35 (100.0%) 0 (0.0%) | 1 [0] 1 (100.0%) 0 (0.0%) | 11 [0] 11 (100.0%) 0 (0.0%) | 23 [0] 23 (100.0%) 0 (0.0%) |
| Currently receiving Phototherapy? | N [N_MISSING_] N (%) No N (%) Yes | 4 [50] 4 (100.0%) 0 (0.0%) |  | 1 [18] 1 (100.0%) 0 (0.0%) | 0 [1] 0 (-%) 0 (-%) | 1 [7] 1 (100.0%) 0 (0.0%) | 0 [10] 0 (-%) 0 (-%) |  | 3 [32] 3 (100.0%) 0 (0.0%) | 0 [1] 0 (-%) 0 (-%) | 1 [10] 1 (100.0%) 0 (0.0%) | 2 [21] 2 (100.0%) 0 (0.0%) |
| Currently using any creams for psoriasis? | N [N_MISSING_] N (%) No N (%) Yes | 4 [50] 1 (25.0%) 3 (75.0%) |  | 1 [18] 0 (0.0%) 1 (100.0%) | 0 [1] 0 (-%) 0 (-%) | 1 [7] 0 (0.0%) 1 (100.0%) | 0 [10] 0 (-%) 0 (-%) |  | 3 [32] 1 (33.3%) 2 (66.7%) | 0 [1] 0 (-%) 0 (-%) | 1 [10] 0 (0.0%) 1 (100.0%) | 2 [21] 1 (50.0%) 1 (50.0%) |
|  | | | | | | | | | | | | |

| **Table 6.1.1:** Use of methotrexate at baseline. Sex-specific figures exclude subjects with no Age or Sex information. | | | | | | | | | | | | |
| --- | --- | --- | --- | --- | --- | --- | --- | --- | --- | --- | --- | --- |
|  |  | All |  | Males | | | |  | Females | | | |
|  |  |  |  | All | < 40 | 40-59 | ≥ 60 |  | All | < 40 | 40-59 | ≥ 60 |
| Currently taking Methotrexate? | N [N_MISSING_] N (%) No N (%) Yes N (%) Not Sure | 331 [13] 178 (53.8%) 149 (45.0%) 4 (1.2%) |  | 120 [2] 75 (62.5%) 43 (35.8%) 2 (1.7%) | 19 [0] 14 (73.7%) 4 (21.1%) 1 (5.3%) | 58 [2] 41 (70.7%) 17 (29.3%) 0 (0.0%) | 43 [0] 20 (46.5%) 22 (51.2%) 1 (2.3%) |  | 198 [3] 95 (48.0%) 102 (51.5%) 1 (0.5%) | 29 [1] 14 (48.3%) 14 (48.3%) 1 (3.4%) | 106 [2] 52 (49.1%) 54 (50.9%) 0 (0.0%) | 63 [0] 29 (46.0%) 34 (54.0%) 0 (0.0%) |
| How are you taking Methotrexate? | N [N_MISSING_] N (%) Tablets N (%) Injections | 149 [0] 120 (80.5%) 29 (19.5%) |  | 43 [0] 38 (88.4%) 5 (11.6%) | 4 [0] 4 (100.0%) 0 (0.0%) | 17 [0] 14 (82.4%) 3 (17.6%) | 22 [0] 20 (90.9%) 2 (9.1%) |  | 102 [0] 79 (77.5%) 23 (22.5%) | 14 [0] 12 (85.7%) 2 (14.3%) | 54 [0] 39 (72.2%) 15 (27.8%) | 34 [0] 28 (82.4%) 6 (17.6%) |
| Weekly dose of Methotrexate | N [N_MISSING_] Mean (SD) [Min, Max] | 146 [3] 16.4 (6.9) [2.5, 30.0] |  | 42 [1] 17.1 (6.8) [2.5, 30.0] | 4 [0] 13.8 (7.8) [2.5, 20.0] | 16 [1] 17.5 (6.7) [2.5, 25.0] | 22 [0] 17.5 (6.9) [2.5, 30.0] |  | 100 [2] 16.2 (7.0) [2.5, 27.5] | 14 [0] 17.0 (6.1) [7.5, 25.0] | 52 [2] 16.7 (7.0) [2.5, 25.0] | 34 [0] 15.0 (7.3) [2.5, 27.5] |
|  | | | | | | | | | | | | |

| **Table 6.1.2:** Use of methotrexate at baseline. Sex-specific figures exclude subjects with no Age or Sex information. Subjects reporting their condition as 'Rheumatoid Arthritis'. | | | | | | | | | | | | |
| --- | --- | --- | --- | --- | --- | --- | --- | --- | --- | --- | --- | --- |
|  |  | All |  | Males | | | |  | Females | | | |
|  |  |  |  | All | < 40 | 40-59 | ≥ 60 |  | All | < 40 | 40-59 | ≥ 60 |
| Currently taking Methotrexate? | N [N_MISSING_] N (%) No N (%) Yes N (%) Not Sure | 190 [1] 71 (37.4%) 118 (62.1%) 1 (0.5%) |  | 55 [0] 20 (36.4%) 34 (61.8%) 1 (1.8%) | 3 [0] 2 (66.7%) 1 (33.3%) 0 (0.0%) | 19 [0] 6 (31.6%) 13 (68.4%) 0 (0.0%) | 33 [0] 12 (36.4%) 20 (60.6%) 1 (3.0%) |  | 133 [1] 50 (37.6%) 83 (62.4%) 0 (0.0%) | 16 [0] 4 (25.0%) 12 (75.0%) 0 (0.0%) | 67 [1] 26 (38.8%) 41 (61.2%) 0 (0.0%) | 50 [0] 20 (40.0%) 30 (60.0%) 0 (0.0%) |
| How are you taking Methotrexate? | N [N_MISSING_] N (%) Tablets N (%) Injections | 118 [0] 97 (82.2%) 21 (17.8%) |  | 34 [0] 29 (85.3%) 5 (14.7%) | 1 [0] 1 (100.0%) 0 (0.0%) | 13 [0] 10 (76.9%) 3 (23.1%) | 20 [0] 18 (90.0%) 2 (10.0%) |  | 83 [0] 67 (80.7%) 16 (19.3%) | 12 [0] 10 (83.3%) 2 (16.7%) | 41 [0] 32 (78.0%) 9 (22.0%) | 30 [0] 25 (83.3%) 5 (16.7%) |
| Weekly dose of Methotrexate | N [N_MISSING_] Mean (SD) [Min, Max] | 116 [2] 16.6 (6.8) [2.5, 30.0] |  | 33 [1] 17.8 (6.7) [2.5, 30.0] | 1 [0] 2.5 (-) [2.5, 2.5] | 12 [1] 18.5 (6.2) [7.5, 25.0] | 20 [0] 18.1 (6.4) [7.5, 30.0] |  | 82 [1] 16.1 (6.8) [2.5, 27.5] | 12 [0] 17.1 (5.5) [7.5, 25.0] | 40 [1] 17.0 (6.7) [2.5, 25.0] | 30 [0] 14.6 (7.3) [2.5, 27.5] |
| . | | | | | | | | | | | | |

| **Table 6.1.3:** Use of methotrexate at baseline. Sex-specific figures exclude subjects with no Age or Sex information. Subjects reporting their condition as 'Psoriasis'. | | | | | | | | | | | | |
| --- | --- | --- | --- | --- | --- | --- | --- | --- | --- | --- | --- | --- |
|  |  | All |  | Males | | | |  | Females | | | |
|  |  |  |  | All | < 40 | 40-59 | ≥ 60 |  | All | < 40 | 40-59 | ≥ 60 |
| Currently taking Methotrexate? | N [N_MISSING_] N (%) No N (%) Yes N (%) Not Sure | 35 [0] 31 (88.6%) 4 (11.4%) 0 (0.0%) |  | 15 [0] 14 (93.3%) 1 (6.7%) 0 (0.0%) | 4 [0] 4 (100.0%) 0 (0.0%) 0 (0.0%) | 10 [0] 9 (90.0%) 1 (10.0%) 0 (0.0%) | 1 [0] 1 (100.0%) 0 (0.0%) 0 (0.0%) |  | 18 [0] 15 (83.3%) 3 (16.7%) 0 (0.0%) | 1 [0] 1 (100.0%) 0 (0.0%) 0 (0.0%) | 12 [0] 9 (75.0%) 3 (25.0%) 0 (0.0%) | 5 [0] 5 (100.0%) 0 (0.0%) 0 (0.0%) |
| How are you taking Methotrexate? | N [N_MISSING_] N (%) Tablets N (%) Injections | 4 [0] 3 (75.0%) 1 (25.0%) |  | 1 [0] 1 (100.0%) 0 (0.0%) | 0 [0] 0 (-%) 0 (-%) | 1 [0] 1 (100.0%) 0 (0.0%) | 0 [0] 0 (-%) 0 (-%) |  | 3 [0] 2 (66.7%) 1 (33.3%) | 0 [0] 0 (-%) 0 (-%) | 3 [0] 2 (66.7%) 1 (33.3%) | 0 [0] 0 (-%) 0 (-%) |
| Weekly dose of Methotrexate | N [N_MISSING_] Mean (SD) [Min, Max] | 4 [0] 16.2 (9.7) [2.5, 25.0] |  | 1 [0] 20.0 (-) [20.0, 20.0] | 0 [-] - (-) [-, -] | 1 [0] 20.0 (-) [20.0, 20.0] | 0 [-] - (-) [-, -] |  | 3 [0] 15.0 (11.5) [2.5, 25.0] | 0 [-] - (-) [-, -] | 3 [0] 15.0 (11.5) [2.5, 25.0] | 0 [-] - (-) [-, -] |
| . | | | | | | | | | | | | |

| **Table 6.1.4:** Use of methotrexate at baseline. Sex-specific figures exclude subjects with no Age or Sex information. Subjects reporting their condition as 'Ankylosing Spondylitis'. | | | | | | | | | | | | |
| --- | --- | --- | --- | --- | --- | --- | --- | --- | --- | --- | --- | --- |
|  |  | All |  | Males | | | |  | Females | | | |
|  |  |  |  | All | < 40 | 40-59 | ≥ 60 |  | All | < 40 | 40-59 | ≥ 60 |
| Currently taking Methotrexate? | N [N_MISSING_] N (%) No N (%) Yes N (%) Not Sure | 43 [0] 38 (88.4%) 3 (7.0%) 2 (4.7%) |  | 32 [0] 29 (90.6%) 2 (6.2%) 1 (3.1%) | 8 [0] 5 (62.5%) 2 (25.0%) 1 (12.5%) | 17 [0] 17 (100.0%) 0 (0.0%) 0 (0.0%) | 7 [0] 7 (100.0%) 0 (0.0%) 0 (0.0%) |  | 11 [0] 9 (81.8%) 1 (9.1%) 1 (9.1%) | 4 [0] 3 (75.0%) 0 (0.0%) 1 (25.0%) | 5 [0] 5 (100.0%) 0 (0.0%) 0 (0.0%) | 2 [0] 1 (50.0%) 1 (50.0%) 0 (0.0%) |
| How are you taking Methotrexate? | N [N_MISSING_] N (%) Tablets N (%) Injections | 3 [0] 3 (100.0%) 0 (0.0%) |  | 2 [0] 2 (100.0%) 0 (0.0%) | 2 [0] 2 (100.0%) 0 (0.0%) | 0 [0] 0 (-%) 0 (-%) | 0 [0] 0 (-%) 0 (-%) |  | 1 [0] 1 (100.0%) 0 (0.0%) | 0 [0] 0 (-%) 0 (-%) | 0 [0] 0 (-%) 0 (-%) | 1 [0] 1 (100.0%) 0 (0.0%) |
| Weekly dose of Methotrexate | N [N_MISSING_] Mean (SD) [Min, Max] | 3 [0] 17.5 (2.5) [15.0, 20.0] |  | 2 [0] 16.2 (1.8) [15.0, 17.5] | 2 [0] 16.2 (1.8) [15.0, 17.5] | 0 [-] - (-) [-, -] | 0 [-] - (-) [-, -] |  | 1 [0] 20.0 (-) [20.0, 20.0] | 0 [-] - (-) [-, -] | 0 [-] - (-) [-, -] | 1 [0] 20.0 (-) [20.0, 20.0] |
|  | | | | | | | | | | | | |

| **Table 6.1.5:** Use of methotrexate at baseline. Sex-specific figures exclude subjects with no Age or Sex information. Subjects reporting their condition as 'Psoriatic Arthritis'. | | | | | | | | | | | | |
| --- | --- | --- | --- | --- | --- | --- | --- | --- | --- | --- | --- | --- |
|  |  | All |  | Males | | | |  | Females | | | |
|  |  |  |  | All | < 40 | 40-59 | ≥ 60 |  | All | < 40 | 40-59 | ≥ 60 |
| Currently taking Methotrexate? | N [N_MISSING_] N (%) No N (%) Yes N (%) Not Sure | 44 [0] 27 (61.4%) 16 (36.4%) 1 (2.3%) |  | 13 [0] 10 (76.9%) 3 (23.1%) 0 (0.0%) | 3 [0] 3 (100.0%) 0 (0.0%) 0 (0.0%) | 9 [0] 7 (77.8%) 2 (22.2%) 0 (0.0%) | 1 [0] 0 (0.0%) 1 (100.0%) 0 (0.0%) |  | 28 [0] 16 (57.1%) 12 (42.9%) 0 (0.0%) | 5 [0] 4 (80.0%) 1 (20.0%) 0 (0.0%) | 18 [0] 10 (55.6%) 8 (44.4%) 0 (0.0%) | 5 [0] 2 (40.0%) 3 (60.0%) 0 (0.0%) |
| How are you taking Methotrexate? | N [N_MISSING_] N (%) Tablets N (%) Injections | 16 [0] 11 (68.8%) 5 (31.2%) |  | 3 [0] 3 (100.0%) 0 (0.0%) | 0 [0] 0 (-%) 0 (-%) | 2 [0] 2 (100.0%) 0 (0.0%) | 1 [0] 1 (100.0%) 0 (0.0%) |  | 12 [0] 7 (58.3%) 5 (41.7%) | 1 [0] 1 (100.0%) 0 (0.0%) | 8 [0] 4 (50.0%) 4 (50.0%) | 3 [0] 2 (66.7%) 1 (33.3%) |
| Weekly dose of Methotrexate | N [N_MISSING_] Mean (SD) [Min, Max] | 16 [0] 15.6 (7.3) [2.5, 25.0] |  | 3 [0] 18.3 (2.9) [15.0, 20.0] | 0 [-] - (-) [-, -] | 2 [0] 17.5 (3.5) [15.0, 20.0] | 1 [0] 20.0 (-) [20.0, 20.0] |  | 12 [0] 15.6 (7.9) [2.5, 25.0] | 1 [0] 7.5 (-) [7.5, 7.5] | 8 [0] 15.9 (8.0) [2.5, 25.0] | 3 [0] 17.5 (9.0) [7.5, 25.0] |
|  | | | | | | | | | | | | |

| **Table 6.2.1:** Use of methotrexate at baseline (only for those completing questionnaires online). Sex-specific figures exclude subjects with no Age or Sex information. | | | | | | | | | | | | |
| --- | --- | --- | --- | --- | --- | --- | --- | --- | --- | --- | --- | --- |
|  |  | All |  | Males | | | |  | Females | | | |
|  |  |  |  | All | < 40 | 40-59 | ≥ 60 |  | All | < 40 | 40-59 | ≥ 60 |
| Currently taking Methotrexate? | N [N_MISSING_] N (%) No N (%) Yes N (%) Not Sure | 277 [13] 154 (55.6%) 120 (43.3%) 3 (1.1%) |  | 101 [2] 69 (68.3%) 31 (30.7%) 1 (1.0%) | 18 [0] 13 (72.2%) 4 (22.2%) 1 (5.6%) | 50 [2] 38 (76.0%) 12 (24.0%) 0 (0.0%) | 33 [0] 18 (54.5%) 15 (45.5%) 0 (0.0%) |  | 163 [3] 77 (47.2%) 85 (52.1%) 1 (0.6%) | 28 [1] 14 (50.0%) 13 (46.4%) 1 (3.6%) | 95 [2] 46 (48.4%) 49 (51.6%) 0 (0.0%) | 40 [0] 17 (42.5%) 23 (57.5%) 0 (0.0%) |
| How are you taking Methotrexate? | N [N_MISSING_] N (%) Tablets N (%) Injections | 120 [0] 96 (80.0%) 24 (20.0%) |  | 31 [0] 27 (87.1%) 4 (12.9%) | 4 [0] 4 (100.0%) 0 (0.0%) | 12 [0] 9 (75.0%) 3 (25.0%) | 15 [0] 14 (93.3%) 1 (6.7%) |  | 85 [0] 66 (77.6%) 19 (22.4%) | 13 [0] 11 (84.6%) 2 (15.4%) | 49 [0] 36 (73.5%) 13 (26.5%) | 23 [0] 19 (82.6%) 4 (17.4%) |
| Weekly dose of Methotrexate | N [N_MISSING_] Mean (SD) [Min, Max] | 119 [1] 16.4 (6.9) [2.5, 27.5] |  | 31 [0] 17.3 (6.9) [2.5, 25.0] | 4 [0] 13.8 (7.8) [2.5, 20.0] | 12 [0] 17.7 (6.8) [2.5, 25.0] | 15 [0] 17.8 (7.1) [2.5, 25.0] |  | 84 [1] 16.1 (6.9) [2.5, 27.5] | 13 [0] 16.7 (6.3) [7.5, 25.0] | 48 [1] 16.4 (6.8) [2.5, 25.0] | 23 [0] 15.3 (7.5) [2.5, 27.5] |
| . | | | | | | | | | | | | |

| **Table 6.3.1:** Use of methotrexate at baseline (only for those completing questionnaires by telephone). Sex-specific figures exclude subjects with no Age or Sex information. | | | | | | | | | | | | |
| --- | --- | --- | --- | --- | --- | --- | --- | --- | --- | --- | --- | --- |
|  |  | All |  | Males | | | |  | Females | | | |
|  |  |  |  | All | < 40 | 40-59 | ≥ 60 |  | All | < 40 | 40-59 | ≥ 60 |
| Currently taking Methotrexate? | N [N_MISSING_] N (%) No N (%) Yes N (%) Not Sure | 54 [0] 24 (44.4%) 29 (53.7%) 1 (1.9%) |  | 19 [0] 6 (31.6%) 12 (63.2%) 1 (5.3%) | 1 [0] 1 (100.0%) 0 (0.0%) 0 (0.0%) | 8 [0] 3 (37.5%) 5 (62.5%) 0 (0.0%) | 10 [0] 2 (20.0%) 7 (70.0%) 1 (10.0%) |  | 35 [0] 18 (51.4%) 17 (48.6%) 0 (0.0%) | 1 [0] 0 (0.0%) 1 (100.0%) 0 (0.0%) | 11 [0] 6 (54.5%) 5 (45.5%) 0 (0.0%) | 23 [0] 12 (52.2%) 11 (47.8%) 0 (0.0%) |
| How are you taking Methotrexate? | N [N_MISSING_] N (%) Tablets N (%) Injections | 29 [0] 24 (82.8%) 5 (17.2%) |  | 12 [0] 11 (91.7%) 1 (8.3%) | 0 [0] 0 (-%) 0 (-%) | 5 [0] 5 (100.0%) 0 (0.0%) | 7 [0] 6 (85.7%) 1 (14.3%) |  | 17 [0] 13 (76.5%) 4 (23.5%) | 1 [0] 1 (100.0%) 0 (0.0%) | 5 [0] 3 (60.0%) 2 (40.0%) | 11 [0] 9 (81.8%) 2 (18.2%) |
| Weekly dose of Methotrexate | N [N_MISSING_] Mean (SD) [Min, Max] | 27 [2] 16.5 (7.3) [2.5, 30.0] |  | 11 [1] 16.8 (6.9) [7.5, 30.0] | 0 [-] - (-) [-, -] | 4 [1] 16.9 (7.5) [7.5, 25.0] | 7 [0] 16.8 (7.2) [10.0, 30.0] |  | 16 [1] 16.2 (7.7) [2.5, 25.0] | 1 [0] 20.0 (-) [20.0, 20.0] | 4 [1] 20.6 (8.8) [7.5, 25.0] | 11 [0] 14.3 (7.3) [2.5, 25.0] |
| . | | | | | | | | | | | | |
