# Supplementary material for: Baseline characteristics and patient reported outcome data of patients prescribed etanercept: web-based and telephone evaluation
Source: BMC Med Res Methodol. 2011 Jun 14;11:91. doi: 10.1186/1471-2288-11-91 (PMC3141801; doi:10.1186/1471-2288-11-91)
Supplement: Additional File 2 — Word document Breakdown of question completion by telephone, internet and all respondents. [file 1471-2288-11-91-S2.DOC]

**Additional File 2**

| **Table 2.1.1.1:** Completed questions at baseline. Sex-specific figures exclude subjects with no Age or Sex information. | | | | | | | | | | | | |
| --- | --- | --- | --- | --- | --- | --- | --- | --- | --- | --- | --- | --- |
|  |  | All |  | Males | | | |  | Females | | | |
|  |  |  |  | All | < 40 | 40-59 | ≥ 60 |  | All | < 40 | 40-59 | ≥ 60 |
| Question 7, Day 1 | N (%) Answered | 344 (100.0%) |  | 122 (100.0%) | 19 (100.0%) | 60 (100.0%) | 43 (100.0%) |  | 201 (100.0%) | 30 (100.0%) | 108 (100.0%) | 63 (100.0%) |
| Question 3, Day 1 | N (%) Answered | 323 (93.9%) |  | 122 (100.0%) | 19 (100.0%) | 60 (100.0%) | 43 (100.0%) |  | 201 (100.0%) | 30 (100.0%) | 108 (100.0%) | 63 (100.0%) |
| Question 2, Day 1 | N (%) Answered | 323 (93.9%) |  | 122 (100.0%) | 19 (100.0%) | 60 (100.0%) | 43 (100.0%) |  | 201 (100.0%) | 30 (100.0%) | 108 (100.0%) | 63 (100.0%) |
| Question 4, Day 1 | N (%) Answered | 336 (97.7%) |  | 122 (100.0%) | 19 (100.0%) | 60 (100.0%) | 43 (100.0%) |  | 201 (100.0%) | 30 (100.0%) | 108 (100.0%) | 63 (100.0%) |
| Question 10 or 13, Day 1 | N (%) Answered | 332 (96.5%) |  | 120 (98.4%) | 19 (100.0%) | 58 (96.7%) | 43 (100.0%) |  | 199 (99.0%) | 29 (96.7%) | 107 (99.1%) | 63 (100.0%) |
| Question 11, Day 1 | N (%) Answered | 331 (96.2%) |  | 120 (98.4%) | 19 (100.0%) | 58 (96.7%) | 43 (100.0%) |  | 198 (98.5%) | 29 (96.7%) | 106 (98.1%) | 63 (100.0%) |
| Question 12, Day 1 | N (%) Answered | 332 (96.5%) |  | 120 (98.4%) | 19 (100.0%) | 58 (96.7%) | 43 (100.0%) |  | 199 (99.0%) | 29 (96.7%) | 107 (99.1%) | 63 (100.0%) |
| Question 35, Day 1 | N (%) Answered | 331 (96.2%) |  | 120 (98.4%) | 19 (100.0%) | 58 (96.7%) | 43 (100.0%) |  | 198 (98.5%) | 29 (96.7%) | 106 (98.1%) | 63 (100.0%) |
| Question 21, Day 1 | N (%) Answered | 330 (95.9%) |  | 120 (98.4%) | 19 (100.0%) | 58 (96.7%) | 43 (100.0%) |  | 198 (98.5%) | 29 (96.7%) | 106 (98.1%) | 63 (100.0%) |
| Question 22, Day 1 | N (%) Answered | 330 (95.9%) |  | 120 (98.4%) | 19 (100.0%) | 58 (96.7%) | 43 (100.0%) |  | 198 (98.5%) | 29 (96.7%) | 106 (98.1%) | 63 (100.0%) |
| Question 23, Day 1 | N (%) Answered | 330 (95.9%) |  | 120 (98.4%) | 19 (100.0%) | 58 (96.7%) | 43 (100.0%) |  | 198 (98.5%) | 29 (96.7%) | 106 (98.1%) | 63 (100.0%) |
| Questions 101-105, Day 1 | N (%) Answered | 319 (92.7%) |  | 116 (95.1%) | 18 (94.7%) | 57 (95.0%) | 41 (95.3%) |  | 192 (95.5%) | 27 (90.0%) | 103 (95.4%) | 62 (98.4%) |

| **Table 2.1.1.2:** Completed questions at baseline. Sex-specific figures exclude subjects with no Age or Sex information. Subjects reporting their condition as 'Rheumatoid Arthritis'. | | | | | | | | | | | | |
| --- | --- | --- | --- | --- | --- | --- | --- | --- | --- | --- | --- | --- |
|  |  | All |  | Males | | | |  | Females | | | |
|  |  |  |  | All | < 40 | 40-59 | ≥ 60 |  | All | < 40 | 40-59 | ≥ 60 |
| Question 7, Day 1 | N (%) Answered | 191 (100.0%) |  | 55 (100.0%) | 3 (100.0%) | 19 (100.0%) | 33 (100.0%) |  | 134 (100.0%) | 16 (100.0%) | 68 (100.0%) | 50 (100.0%) |
| Question 3, Day 1 | N (%) Answered | 189 (99.0%) |  | 55 (100.0%) | 3 (100.0%) | 19 (100.0%) | 33 (100.0%) |  | 134 (100.0%) | 16 (100.0%) | 68 (100.0%) | 50 (100.0%) |
| Question 2, Day 1 | N (%) Answered | 189 (99.0%) |  | 55 (100.0%) | 3 (100.0%) | 19 (100.0%) | 33 (100.0%) |  | 134 (100.0%) | 16 (100.0%) | 68 (100.0%) | 50 (100.0%) |
| Question 4, Day 1 | N (%) Answered | 191 (100.0%) |  | 55 (100.0%) | 3 (100.0%) | 19 (100.0%) | 33 (100.0%) |  | 134 (100.0%) | 16 (100.0%) | 68 (100.0%) | 50 (100.0%) |
| Question 10 or 13, Day 1 | N (%) Answered | 191 (100.0%) |  | 55 (100.0%) | 3 (100.0%) | 19 (100.0%) | 33 (100.0%) |  | 134 (100.0%) | 16 (100.0%) | 68 (100.0%) | 50 (100.0%) |
| Question 11, Day 1 | N (%) Answered | 190 (99.5%) |  | 55 (100.0%) | 3 (100.0%) | 19 (100.0%) | 33 (100.0%) |  | 133 (99.3%) | 16 (100.0%) | 67 (98.5%) | 50 (100.0%) |
| Question 12, Day 1 | N (%) Answered | 191 (100.0%) |  | 55 (100.0%) | 3 (100.0%) | 19 (100.0%) | 33 (100.0%) |  | 134 (100.0%) | 16 (100.0%) | 68 (100.0%) | 50 (100.0%) |
| Question 35, Day 1 | N (%) Answered | 190 (99.5%) |  | 55 (100.0%) | 3 (100.0%) | 19 (100.0%) | 33 (100.0%) |  | 133 (99.3%) | 16 (100.0%) | 67 (98.5%) | 50 (100.0%) |
| Question 21, Day 1 | N (%) Answered | 190 (99.5%) |  | 55 (100.0%) | 3 (100.0%) | 19 (100.0%) | 33 (100.0%) |  | 133 (99.3%) | 16 (100.0%) | 67 (98.5%) | 50 (100.0%) |
| Question 22, Day 1 | N (%) Answered | 190 (99.5%) |  | 55 (100.0%) | 3 (100.0%) | 19 (100.0%) | 33 (100.0%) |  | 133 (99.3%) | 16 (100.0%) | 67 (98.5%) | 50 (100.0%) |
| Question 23, Day 1 | N (%) Answered | 190 (99.5%) |  | 55 (100.0%) | 3 (100.0%) | 19 (100.0%) | 33 (100.0%) |  | 133 (99.3%) | 16 (100.0%) | 67 (98.5%) | 50 (100.0%) |
| Questions 101-105, Day 1 | N (%) Answered | 181 (94.8%) |  | 51 (92.7%) | 2 (66.7%) | 18 (94.7%) | 31 (93.9%) |  | 129 (96.3%) | 15 (93.8%) | 65 (95.6%) | 49 (98.0%) |

| **Table 2.1.1.3:** Completed questions at baseline. Sex-specific figures exclude subjects with no Age or Sex information. Subjects reporting their condition as 'Psoriasis'. | | | | | | | | | | | | |
| --- | --- | --- | --- | --- | --- | --- | --- | --- | --- | --- | --- | --- |
|  |  | All |  | Males | | | |  | Females | | | |
|  |  |  |  | All | < 40 | 40-59 | ≥ 60 |  | All | < 40 | 40-59 | ≥ 60 |
| Question 7, Day 1 | N (%) Answered | 35 (100.0%) |  | 15 (100.0%) | 4 (100.0%) | 10 (100.0%) | 1 (100.0%) |  | 18 (100.0%) | 1 (100.0%) | 12 (100.0%) | 5 (100.0%) |
| Question 3, Day 1 | N (%) Answered | 33 (94.3%) |  | 15 (100.0%) | 4 (100.0%) | 10 (100.0%) | 1 (100.0%) |  | 18 (100.0%) | 1 (100.0%) | 12 (100.0%) | 5 (100.0%) |
| Question 2, Day 1 | N (%) Answered | 33 (94.3%) |  | 15 (100.0%) | 4 (100.0%) | 10 (100.0%) | 1 (100.0%) |  | 18 (100.0%) | 1 (100.0%) | 12 (100.0%) | 5 (100.0%) |
| Question 4, Day 1 | N (%) Answered | 35 (100.0%) |  | 15 (100.0%) | 4 (100.0%) | 10 (100.0%) | 1 (100.0%) |  | 18 (100.0%) | 1 (100.0%) | 12 (100.0%) | 5 (100.0%) |
| Question 10 or 13, Day 1 | N (%) Answered | 35 (100.0%) |  | 15 (100.0%) | 4 (100.0%) | 10 (100.0%) | 1 (100.0%) |  | 18 (100.0%) | 1 (100.0%) | 12 (100.0%) | 5 (100.0%) |
| Question 11, Day 1 | N (%) Answered | 35 (100.0%) |  | 15 (100.0%) | 4 (100.0%) | 10 (100.0%) | 1 (100.0%) |  | 18 (100.0%) | 1 (100.0%) | 12 (100.0%) | 5 (100.0%) |
| Question 12, Day 1 | N (%) Answered | 35 (100.0%) |  | 15 (100.0%) | 4 (100.0%) | 10 (100.0%) | 1 (100.0%) |  | 18 (100.0%) | 1 (100.0%) | 12 (100.0%) | 5 (100.0%) |
| Question 35, Day 1 | N (%) Answered | 35 (100.0%) |  | 15 (100.0%) | 4 (100.0%) | 10 (100.0%) | 1 (100.0%) |  | 18 (100.0%) | 1 (100.0%) | 12 (100.0%) | 5 (100.0%) |
| Question 21, Day 1 | N (%) Answered | 35 (100.0%) |  | 15 (100.0%) | 4 (100.0%) | 10 (100.0%) | 1 (100.0%) |  | 18 (100.0%) | 1 (100.0%) | 12 (100.0%) | 5 (100.0%) |
| Question 22, Day 1 | N (%) Answered | 35 (100.0%) |  | 15 (100.0%) | 4 (100.0%) | 10 (100.0%) | 1 (100.0%) |  | 18 (100.0%) | 1 (100.0%) | 12 (100.0%) | 5 (100.0%) |
| Question 23, Day 1 | N (%) Answered | 35 (100.0%) |  | 15 (100.0%) | 4 (100.0%) | 10 (100.0%) | 1 (100.0%) |  | 18 (100.0%) | 1 (100.0%) | 12 (100.0%) | 5 (100.0%) |
| Questions 101-105, Day 1 | N (%) Answered | 34 (97.1%) |  | 15 (100.0%) | 4 (100.0%) | 10 (100.0%) | 1 (100.0%) |  | 17 (94.4%) | 1 (100.0%) | 11 (91.7%) | 5 (100.0%) |

| **Table 2.1.1.4:** Completed questions at baseline. Sex-specific figures exclude subjects with no Age or Sex information. Subjects reporting their condition as 'Ankylosing Spondylitis'. | | | | | | | | | | | | |
| --- | --- | --- | --- | --- | --- | --- | --- | --- | --- | --- | --- | --- |
|  |  | All |  | Males | | | |  | Females | | | |
|  |  |  |  | All | < 40 | 40-59 | ≥ 60 |  | All | < 40 | 40-59 | ≥ 60 |
| Question 7, Day 1 | N (%) Answered | 43 (100.0%) |  | 32 (100.0%) | 8 (100.0%) | 17 (100.0%) | 7 (100.0%) |  | 11 (100.0%) | 4 (100.0%) | 5 (100.0%) | 2 (100.0%) |
| Question 3, Day 1 | N (%) Answered | 43 (100.0%) |  | 32 (100.0%) | 8 (100.0%) | 17 (100.0%) | 7 (100.0%) |  | 11 (100.0%) | 4 (100.0%) | 5 (100.0%) | 2 (100.0%) |
| Question 2, Day 1 | N (%) Answered | 43 (100.0%) |  | 32 (100.0%) | 8 (100.0%) | 17 (100.0%) | 7 (100.0%) |  | 11 (100.0%) | 4 (100.0%) | 5 (100.0%) | 2 (100.0%) |
| Question 4, Day 1 | N (%) Answered | 43 (100.0%) |  | 32 (100.0%) | 8 (100.0%) | 17 (100.0%) | 7 (100.0%) |  | 11 (100.0%) | 4 (100.0%) | 5 (100.0%) | 2 (100.0%) |
| Question 10 or 13, Day 1 | N (%) Answered | 43 (100.0%) |  | 32 (100.0%) | 8 (100.0%) | 17 (100.0%) | 7 (100.0%) |  | 11 (100.0%) | 4 (100.0%) | 5 (100.0%) | 2 (100.0%) |
| Question 11, Day 1 | N (%) Answered | 43 (100.0%) |  | 32 (100.0%) | 8 (100.0%) | 17 (100.0%) | 7 (100.0%) |  | 11 (100.0%) | 4 (100.0%) | 5 (100.0%) | 2 (100.0%) |
| Question 12, Day 1 | N (%) Answered | 43 (100.0%) |  | 32 (100.0%) | 8 (100.0%) | 17 (100.0%) | 7 (100.0%) |  | 11 (100.0%) | 4 (100.0%) | 5 (100.0%) | 2 (100.0%) |
| Question 35, Day 1 | N (%) Answered | 43 (100.0%) |  | 32 (100.0%) | 8 (100.0%) | 17 (100.0%) | 7 (100.0%) |  | 11 (100.0%) | 4 (100.0%) | 5 (100.0%) | 2 (100.0%) |
| Question 21, Day 1 | N (%) Answered | 43 (100.0%) |  | 32 (100.0%) | 8 (100.0%) | 17 (100.0%) | 7 (100.0%) |  | 11 (100.0%) | 4 (100.0%) | 5 (100.0%) | 2 (100.0%) |
| Question 22, Day 1 | N (%) Answered | 43 (100.0%) |  | 32 (100.0%) | 8 (100.0%) | 17 (100.0%) | 7 (100.0%) |  | 11 (100.0%) | 4 (100.0%) | 5 (100.0%) | 2 (100.0%) |
| Question 23, Day 1 | N (%) Answered | 43 (100.0%) |  | 32 (100.0%) | 8 (100.0%) | 17 (100.0%) | 7 (100.0%) |  | 11 (100.0%) | 4 (100.0%) | 5 (100.0%) | 2 (100.0%) |
| Questions 101-105, Day 1 | N (%) Answered | 43 (100.0%) |  | 32 (100.0%) | 8 (100.0%) | 17 (100.0%) | 7 (100.0%) |  | 11 (100.0%) | 4 (100.0%) | 5 (100.0%) | 2 (100.0%) |

| **Table 2.1.1.5:** Completed questions at baseline. Sex-specific figures exclude subjects with no Age or Sex information. Subjects reporting their condition as 'Psoriatic Arthritis'. | | | | | | | | | | | | |
| --- | --- | --- | --- | --- | --- | --- | --- | --- | --- | --- | --- | --- |
|  |  | All |  | Males | | | |  | Females | | | |
|  |  |  |  | All | < 40 | 40-59 | ≥ 60 |  | All | < 40 | 40-59 | ≥ 60 |
| Question 7, Day 1 | N (%) Answered | 44 (100.0%) |  | 13 (100.0%) | 3 (100.0%) | 9 (100.0%) | 1 (100.0%) |  | 28 (100.0%) | 5 (100.0%) | 18 (100.0%) | 5 (100.0%) |
| Question 3, Day 1 | N (%) Answered | 41 (93.2%) |  | 13 (100.0%) | 3 (100.0%) | 9 (100.0%) | 1 (100.0%) |  | 28 (100.0%) | 5 (100.0%) | 18 (100.0%) | 5 (100.0%) |
| Question 2, Day 1 | N (%) Answered | 41 (93.2%) |  | 13 (100.0%) | 3 (100.0%) | 9 (100.0%) | 1 (100.0%) |  | 28 (100.0%) | 5 (100.0%) | 18 (100.0%) | 5 (100.0%) |
| Question 4, Day 1 | N (%) Answered | 44 (100.0%) |  | 13 (100.0%) | 3 (100.0%) | 9 (100.0%) | 1 (100.0%) |  | 28 (100.0%) | 5 (100.0%) | 18 (100.0%) | 5 (100.0%) |
| Question 10 or 13, Day 1 | N (%) Answered | 44 (100.0%) |  | 13 (100.0%) | 3 (100.0%) | 9 (100.0%) | 1 (100.0%) |  | 28 (100.0%) | 5 (100.0%) | 18 (100.0%) | 5 (100.0%) |
| Question 11, Day 1 | N (%) Answered | 44 (100.0%) |  | 13 (100.0%) | 3 (100.0%) | 9 (100.0%) | 1 (100.0%) |  | 28 (100.0%) | 5 (100.0%) | 18 (100.0%) | 5 (100.0%) |
| Question 12, Day 1 | N (%) Answered | 44 (100.0%) |  | 13 (100.0%) | 3 (100.0%) | 9 (100.0%) | 1 (100.0%) |  | 28 (100.0%) | 5 (100.0%) | 18 (100.0%) | 5 (100.0%) |
| Question 35, Day 1 | N (%) Answered | 44 (100.0%) |  | 13 (100.0%) | 3 (100.0%) | 9 (100.0%) | 1 (100.0%) |  | 28 (100.0%) | 5 (100.0%) | 18 (100.0%) | 5 (100.0%) |
| Question 21, Day 1 | N (%) Answered | 43 (97.7%) |  | 13 (100.0%) | 3 (100.0%) | 9 (100.0%) | 1 (100.0%) |  | 28 (100.0%) | 5 (100.0%) | 18 (100.0%) | 5 (100.0%) |
| Question 22, Day 1 | N (%) Answered | 43 (97.7%) |  | 13 (100.0%) | 3 (100.0%) | 9 (100.0%) | 1 (100.0%) |  | 28 (100.0%) | 5 (100.0%) | 18 (100.0%) | 5 (100.0%) |
| Question 23, Day 1 | N (%) Answered | 43 (97.7%) |  | 13 (100.0%) | 3 (100.0%) | 9 (100.0%) | 1 (100.0%) |  | 28 (100.0%) | 5 (100.0%) | 18 (100.0%) | 5 (100.0%) |
| Questions 101-105, Day 1 | N (%) Answered | 43 (97.7%) |  | 13 (100.0%) | 3 (100.0%) | 9 (100.0%) | 1 (100.0%) |  | 28 (100.0%) | 5 (100.0%) | 18 (100.0%) | 5 (100.0%) |

| **Table 2.1.2.1:** Completed questions at baseline (only for those completing questionnaires online). Sex-specific figures exclude subjects with no Age or Sex information. | | | | | | | | | | | | |
| --- | --- | --- | --- | --- | --- | --- | --- | --- | --- | --- | --- | --- |
|  |  | All |  | Males | | | |  | Females | | | |
|  |  |  |  | All | < 40 | 40-59 | ≥ 60 |  | All | < 40 | 40-59 | ≥ 60 |
| Question 7, Day 1 | N (%) Answered | 290 (100.0%) |  | 103 (100.0%) | 18 (100.0%) | 52 (100.0%) | 33 (100.0%) |  | 166 (100.0%) | 29 (100.0%) | 97 (100.0%) | 40 (100.0%) |
| Question 3, Day 1 | N (%) Answered | 269 (92.8%) |  | 103 (100.0%) | 18 (100.0%) | 52 (100.0%) | 33 (100.0%) |  | 166 (100.0%) | 29 (100.0%) | 97 (100.0%) | 40 (100.0%) |
| Question 2, Day 1 | N (%) Answered | 269 (92.8%) |  | 103 (100.0%) | 18 (100.0%) | 52 (100.0%) | 33 (100.0%) |  | 166 (100.0%) | 29 (100.0%) | 97 (100.0%) | 40 (100.0%) |
| Question 4, Day 1 | N (%) Answered | 282 (97.2%) |  | 103 (100.0%) | 18 (100.0%) | 52 (100.0%) | 33 (100.0%) |  | 166 (100.0%) | 29 (100.0%) | 97 (100.0%) | 40 (100.0%) |
| Question 10 or 13, Day 1 | N (%) Answered | 278 (95.9%) |  | 101 (98.1%) | 18 (100.0%) | 50 (96.2%) | 33 (100.0%) |  | 164 (98.8%) | 28 (96.6%) | 96 (99.0%) | 40 (100.0%) |
| Question 11, Day 1 | N (%) Answered | 277 (95.5%) |  | 101 (98.1%) | 18 (100.0%) | 50 (96.2%) | 33 (100.0%) |  | 163 (98.2%) | 28 (96.6%) | 95 (97.9%) | 40 (100.0%) |
| Question 12, Day 1 | N (%) Answered | 278 (95.9%) |  | 101 (98.1%) | 18 (100.0%) | 50 (96.2%) | 33 (100.0%) |  | 164 (98.8%) | 28 (96.6%) | 96 (99.0%) | 40 (100.0%) |
| Question 35, Day 1 | N (%) Answered | 277 (95.5%) |  | 101 (98.1%) | 18 (100.0%) | 50 (96.2%) | 33 (100.0%) |  | 163 (98.2%) | 28 (96.6%) | 95 (97.9%) | 40 (100.0%) |
| Question 21, Day 1 | N (%) Answered | 276 (95.2%) |  | 101 (98.1%) | 18 (100.0%) | 50 (96.2%) | 33 (100.0%) |  | 163 (98.2%) | 28 (96.6%) | 95 (97.9%) | 40 (100.0%) |
| Question 22, Day 1 | N (%) Answered | 276 (95.2%) |  | 101 (98.1%) | 18 (100.0%) | 50 (96.2%) | 33 (100.0%) |  | 163 (98.2%) | 28 (96.6%) | 95 (97.9%) | 40 (100.0%) |
| Question 23, Day 1 | N (%) Answered | 276 (95.2%) |  | 101 (98.1%) | 18 (100.0%) | 50 (96.2%) | 33 (100.0%) |  | 163 (98.2%) | 28 (96.6%) | 95 (97.9%) | 40 (100.0%) |
| Questions 101-105, Day 1 | N (%) Answered | 265 (91.4%) |  | 97 (94.2%) | 17 (94.4%) | 49 (94.2%) | 31 (93.9%) |  | 157 (94.6%) | 26 (89.7%) | 92 (94.8%) | 39 (97.5%) |

| **Table 2.1.3.1:** Completed questions at baseline (only for those completing questionnaires by telephone). Sex-specific figures exclude subjects with no Age or Sex information. | | | | | | | | | | | | |
| --- | --- | --- | --- | --- | --- | --- | --- | --- | --- | --- | --- | --- |
|  |  | All |  | Males | | | |  | Females | | | |
|  |  |  |  | All | < 40 | 40-59 | ≥ 60 |  | All | < 40 | 40-59 | ≥ 60 |
| Question 7, Day 1 | N (%) Answered | 290 (100.0%) |  | 103 (100.0%) | 18 (100.0%) | 52 (100.0%) | 33 (100.0%) |  | 166 (100.0%) | 29 (100.0%) | 97 (100.0%) | 40 (100.0%) |
| Question 3, Day 1 | N (%) Answered | 269 (92.8%) |  | 103 (100.0%) | 18 (100.0%) | 52 (100.0%) | 33 (100.0%) |  | 166 (100.0%) | 29 (100.0%) | 97 (100.0%) | 40 (100.0%) |
| Question 2, Day 1 | N (%) Answered | 269 (92.8%) |  | 103 (100.0%) | 18 (100.0%) | 52 (100.0%) | 33 (100.0%) |  | 166 (100.0%) | 29 (100.0%) | 97 (100.0%) | 40 (100.0%) |
| Question 4, Day 1 | N (%) Answered | 282 (97.2%) |  | 103 (100.0%) | 18 (100.0%) | 52 (100.0%) | 33 (100.0%) |  | 166 (100.0%) | 29 (100.0%) | 97 (100.0%) | 40 (100.0%) |
| Question 10 or 13, Day 1 | N (%) Answered | 278 (95.9%) |  | 101 (98.1%) | 18 (100.0%) | 50 (96.2%) | 33 (100.0%) |  | 164 (98.8%) | 28 (96.6%) | 96 (99.0%) | 40 (100.0%) |
| Question 11, Day 1 | N (%) Answered | 277 (95.5%) |  | 101 (98.1%) | 18 (100.0%) | 50 (96.2%) | 33 (100.0%) |  | 163 (98.2%) | 28 (96.6%) | 95 (97.9%) | 40 (100.0%) |
| Question 12, Day 1 | N (%) Answered | 278 (95.9%) |  | 101 (98.1%) | 18 (100.0%) | 50 (96.2%) | 33 (100.0%) |  | 164 (98.8%) | 28 (96.6%) | 96 (99.0%) | 40 (100.0%) |
| Question 35, Day 1 | N (%) Answered | 277 (95.5%) |  | 101 (98.1%) | 18 (100.0%) | 50 (96.2%) | 33 (100.0%) |  | 163 (98.2%) | 28 (96.6%) | 95 (97.9%) | 40 (100.0%) |
| Question 21, Day 1 | N (%) Answered | 276 (95.2%) |  | 101 (98.1%) | 18 (100.0%) | 50 (96.2%) | 33 (100.0%) |  | 163 (98.2%) | 28 (96.6%) | 95 (97.9%) | 40 (100.0%) |
| Question 22, Day 1 | N (%) Answered | 276 (95.2%) |  | 101 (98.1%) | 18 (100.0%) | 50 (96.2%) | 33 (100.0%) |  | 163 (98.2%) | 28 (96.6%) | 95 (97.9%) | 40 (100.0%) |
| Question 23, Day 1 | N (%) Answered | 276 (95.2%) |  | 101 (98.1%) | 18 (100.0%) | 50 (96.2%) | 33 (100.0%) |  | 163 (98.2%) | 28 (96.6%) | 95 (97.9%) | 40 (100.0%) |
| Questions 101-105, Day 1 | N (%) Answered | 265 (91.4%) |  | 97 (94.2%) | 17 (94.4%) | 49 (94.2%) | 31 (93.9%) |  | 157 (94.6%) | 26 (89.7%) | 92 (94.8%) | 39 (97.5%) |
